# Reference programme: diagnosis and treatment of headache disorders and facial pain. Danish Headache Society, 3rd edition, 2020

**DOI:** 10.1186/s10194-021-01228-4

**Published:** 2021-04-08

**Authors:** Henrik W. Schytz, Faisal M. Amin, Rigmor H. Jensen, Louise Carlsen, Stine Maarbjerg, Nunu Lund, Karen Aegidius, Lise L. Thomsen, Flemming W. Bach, Dagmar Beier, Hanne Johansen, Jakob M. Hansen, Helge Kasch, Signe B. Munksgaard, Lars Poulsen, Per Schmidt Sørensen, Peter T. Schmidt-Hansen, Vlasta V. Cvetkovic, Messoud Ashina, Lars Bendtsen

**Affiliations:** 1 Danish Headache Center, Department of Neurology, Rigshospitalet-Glostrup, Faculty of Health and Medical Sciences, University of Copenhagen, Valdemar Hansen Vej 5, 2600 Glostrup, Denmark; 2 Department of Neurology, Odense University Hospital, Odense, Denmark; 3 Specialized Pediatric Clinic, Jægersborgvej 66B, 2. Sal, 2800 Kgs. Lyngby, Denmark; 4 Department of Neurology, Aarhus University Hospital, Aarhus, Denmark; 5 The Migraine and Headache Association (https://www.hovedpineforeningen.dk), Toftehøj 90, 6470 Sydals, Denmark; 6 National Headache Knowledge Center, Danish Headache Center, Rigshospitalet-Glostrup, Valdemar Hansen Vej 5, Glostrup, 2600 Denmark; 7 Department of Neurology, Spinal Cord Injury Centre of Western Denmark, Viborg Hospital, Viborg, Denmark; 8 General Practice, Clinic Laegehuset Nr. Broby, Saksenballe 5, 5672 Broby, Denmark; 9 Neurological Specialist Clinic, Ny Tilemannsvej 4, 8450 Hammel, Denmark; 10 Neurological Specialist Clinic, Idrætsvej 101, 2650 Hvidovre, Denmark

## Abstract

Headache and facial pain are among the most common, disabling and costly diseases in Europe, which demands for high quality health care on all levels within the health system. The role of the Danish Headache Society is to educate and advocate for the needs of patients with headache and facial pain. Therefore, the Danish Headache Society has launched a third version of the guideline for the diagnosis, organization and treatment of the most common types of headaches and facial pain in Denmark. The second edition was published in Danish in 2010 and has been a great success, but as new knowledge and treatments have emerged it was timely to revise the guideline. The recommendations for the primary headaches and facial pain are largely in accordance with the European guidelines produced by the European Academy of Neurology. The guideline should be used a practical tool for use in daily clinical practice for primary care physicians, neurologists with a common interest in headache, as well as other health-care professionals treating headache patients. The guideline first describes how to examine and diagnose the headache patient and how headache treatment is organized in Denmark. This description is followed by sections on the characteristics, diagnosis and treatment of each of the most common primary and secondary headache disorders and trigeminal neuralgia. The guideline includes many tables to facilitate a quick overview. Finally, the particular challenges regarding migraine and female hormones as well as headache in children are addressed.

## Introduction

Headache diseases, especially migraine, are at the very top of the WHO’s list of the most disabling diseases, especially in the most productive years of young adulthood [1]. Every third Dane have at some point in their life sought a doctor due to a headache. In an average general practice in Denmark, more than 10% of patients have migraine and 5% have a chronic headache. Due to the frequency, the total socio-economic costs of headaches are extensive and headache diseases account for 20% of the total sickness absence in the Danish labour market. Loss of quality of life for the person suffering from headaches and their family is also significant. Medication consumption is increasing and with the introduction of new specific preventive migraine medications, there is a need for an updated treatment strategy in Denmark. The vast majority of Danes suffering from headache are treated in the primary sector and should for the most part continue to be treated there in the future, but there is an increasing need for clear guidelines for examining and organizing specialist treatment of severe and rare headache conditions.

There are international guidelines and general recommendations for the treatment of migraines and other primary headache diseases. It is important that these guidelines are implemented and adjusted to Danish conditions.

Neurological specialists in Denmark typically treat patients with trigeminal neuralgia and other types of facial pain, but here too there is an increasing need for the organization and systematization of treatment options. This type of pain condition is often taken care of by headache specialists and is therefore included in the current guidelines.

Based on this, the Danish Headache Society created a working committee to update the Danish reference program for headache diseases and facial pain from 2010. The current reference program has been created in adherence to the general recommendations from the National Board of Health’s previous reference program committee and has been through a hearing phase at the Danish Neurological Society and the Danish Headache Society. In the present paper, the reference programme is translated into English and adapted to journal format. The original reference program in Danish can be downloaded at https://dhos.dk/wp-content/uploads/2020/06/2932-Referenceprogram_2020_final_web-24.06.20.pdf.

### Objective

The objective is to create common guidelines for diagnosing, organizing and treating the most common primary headache diseases such as migraines, tension-type headache and cluster headache as well as trigeminal neuralgia in Denmark, as well as describe important warning signs of serious life-threatening and other secondary headache conditions.

### Introduction to the guidelines

The current guideline includes tables in order to promote user-friendliness. The individual Sections can therefore be read separately, where the most important points are repeated. The tables list ICHD-3 and ICD-10 diagnoses. Following an introductory Section with background and general information as well as a summary, the focus is on the diagnostic process, investigation and organisation. In the headache disorders and facial pain, the medical history is very important, as in the vast majority of cases the diagnosis is primarily made based on the medical history. A correct diagnosis is essential for a correct treatment.

Next, the individual headache diseases are described in separate Sections with characteristics, diagnosis, differential diagnoses and treatment. Finally, we describe the specific problem-areas related to headache in children and in women in relation to hormones, pregnancy and lactation.

There are comprehensive evidence assessments of the recommended treatment, but it has been deliberately chosen not to state the level of evidence class in order to improve readability and reduce the magnitude of information presented. Kindly refer to the recommended literature for further information.

### Target audience

All physicians and other health-care professionals who see patients with headaches and facial pain, in particular general practitioners, physicians in training, neurologists and paediatricians in private practice and hospitals as well as decision makers in the public sector.

## Diagnosis and organization

### Occurrence

Most people have experienced headaches occasionally, and consider this a normal, transient phenomenon. However, headache is a problem for approximately 40% of the European population. In an average general practice (1500 patients) there are on average:

- 150 adults and 30 children with bothersome migraines.
- 65 adults and 10 children with daily headaches and a large proportion of these have medication overuse headache.
- One patient with cluster headache.

Table 1 summarizes the most common types of headache and facial pain.

**Table 1:** The most important types of headache and facial pain

Type	Probable diagnosis	Description
Acute headache	Subarachnoid haemorrhage, and others.	Acute onset, severe headache +/− neurological symptoms
Episodic Headache	Migraine +/− aura	Pulsating headache, aggravation by physical activity with nausea, phono- and photophobia
	Tension-type headache	Pressure headache without associated symptoms
	Cluster headache and others	Unilateral headache with ipsilateral autonomic facial symptoms
	Trigeminal neuralgia	Seconds lasting unilateral severe
Chronic headache	Chronic tension-type headache	Pressure headache without associated symptoms or medication overuse headache
	Medication-overuse headache	Use of acute pain medication more than 10–15 days per month
	Intracranial hypertension, incl. Brain tumor headache	Frequent and increasing headache with nausea and neurological symptoms

The individual patient can suffer from different types of headache and facial pain. There are several headache disorders, which are secondary to other diseases. Some of these secondary headaches are serious, but generally these are rare and less than 1% of all headache patients in primary care (see section “Secondary headaches”)

### Taking a medical history on headache or facial pain

The medical history is crucial in the diagnosis of all primary headache conditions, facial pain and in the case of medication overuse medication (Table 2). There are no definite diagnostic tests. The patient’s medical history should clarify any warning signs of a serious secondary headache. Warning signals, in the medical history or the physical examination, which warrant further examination, are (see also Section 7 “Secondary forms of headache”):

- New onset headache
- Thunderclap headache (sudden onset of severe headache)
- Sudden headache occurring during strenuous physical or sexual activity
- Headache with atypical aura (lasts over 1 h or includes motor outcomes)
- Headache with aura developed while using birth control pills
- New onset of headache in a patient with cancer or HIV infection
- Headache accompanied by fever
- Headache accompanied by neurological outcomes phrased migraine aura
- Progressive headache over weeks
- New onset headache in patients under 10 years of age or over 40 years of age
- Headache, which is position-dependent

**Table:** Table 2

Useful questions	
How many different types of headache/facial pain do you have? Separate history must be taken for each type!	
Time course	When did the headache start? How frequent is the headache (episodic, daily and/or constant)? Duration of each attack (seconds/minutes/hours/days)?
Character	Pain intensity? Pain quality and type? Where is the pain located and does the pain move? Associated symptoms?
Reasons	Trigger factors and/or dispositions? Aggravating or soothing factors? Family dispositions for headache / facial pain?
Ictal behavior	What do you do during the attack? How does the attack affect your activity level? Medication intake, if yes: which and what dose?
General health state interictally	With or without any symptoms between attacks? Worries of anxiety for new attacks and/or their reasons?

### Physical examination of a headache/facial pain patient

Physical and neurological examination is performed to rule out or confirm secondary headaches. The physical examination in an otherwise healthy patient with a primary headache will most often be normal. During an attack of cluster headache, there are possible physical findings such as tearing, reddening of the eye and ptosis. In trigeminal neuralgia, trigger points for the pain can most often be identified. Blood pressure and heart rate should always be investigated. CT / MRI scan is most often not indicated in a patient with a long history of headache, but should be performed if the history or physical examination is unclear or indicates that the headache is due to secondary condition. MRI scan is always indicated for facial pain.

### Diagnostic diary and calendar

**Fig. 1:**
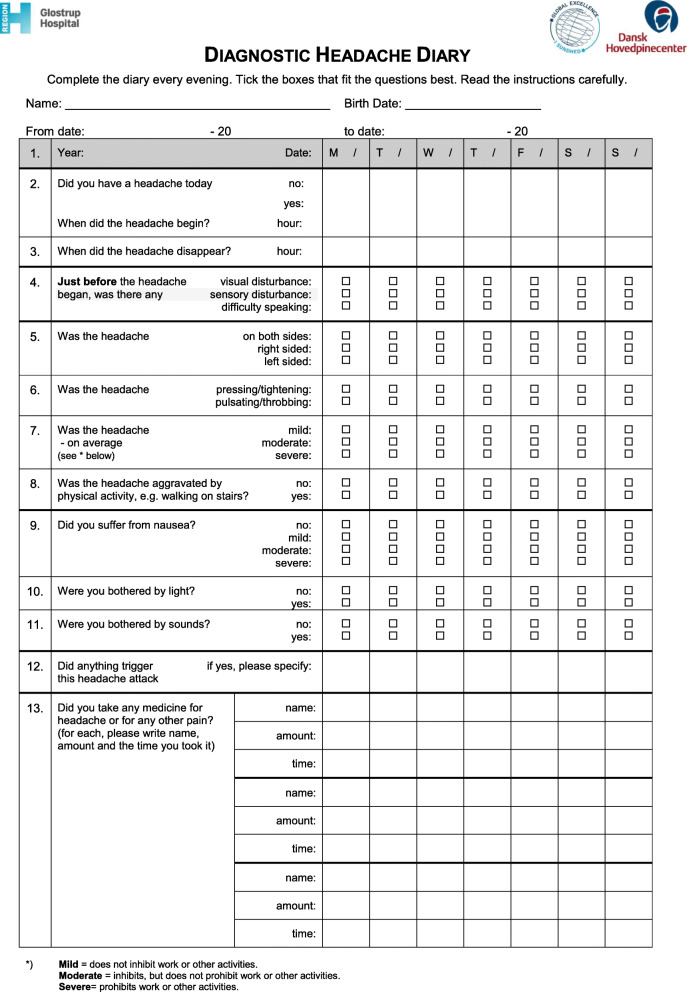
Diagnostic headache diary

**Fig. 2:**
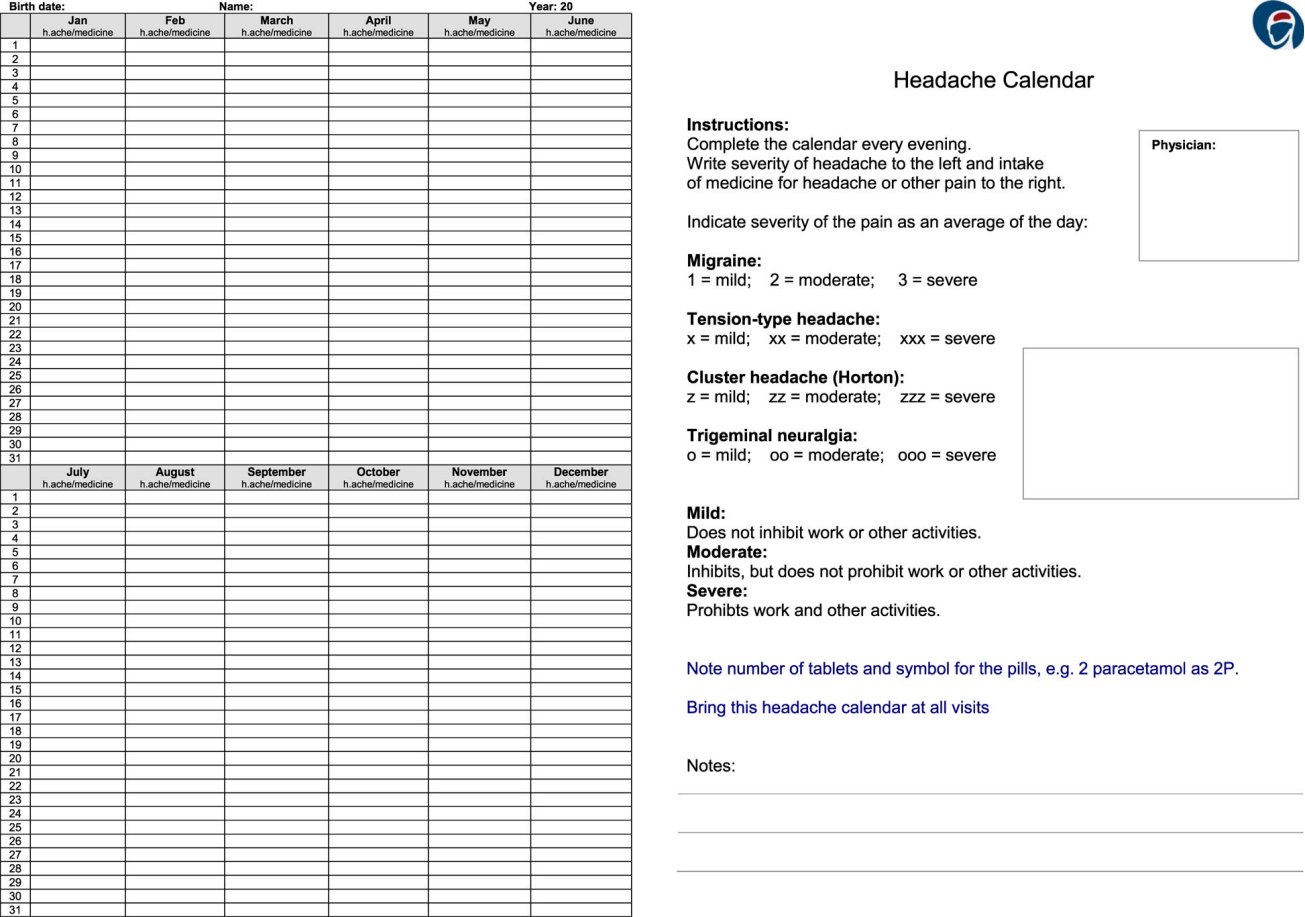
Headache calender

When serious secondary headache has been excluded, it is recommended to use a headache diary (Fig. 1) for at least 4 weeks and a headache calendar (Fig. 2) for a few months. Headache diaries and calendars can be downloaded from the Danish Headache Society website www.dhos.dk. These tools can reveal the headache pattern to the physician and patient, and thereby assist in identifying trigger factors and headache medication overuse.

### Organization of treatment in Denmark

**Fig. 3:**
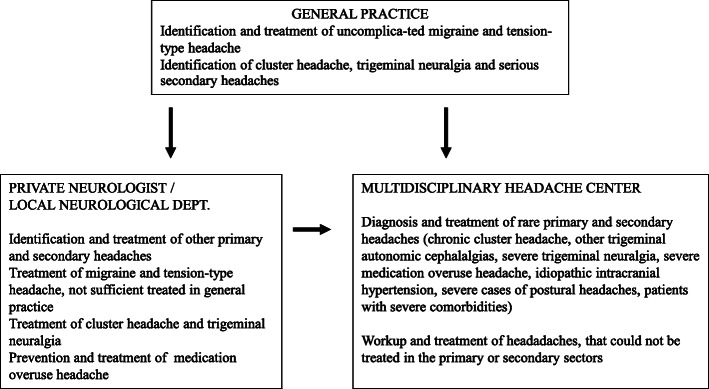
Organisation of treatment in Denmark

Investigation and treatment are generally performed at three levels (see Fig. 3). In Denmark, diagnosis and treatment of headaches is, and should in general be, performed by the GP, i.e. in the primary health care.

At the second level, diagnosis and treatment are handled by the practicing specialist of neurology or other related professionals with interest and experience in pain conditions or by local neurological departments.

The tertiary level consists of headache centres, where medical specialists and interdisciplinary staff specialized in headache conditions are responsible for diagnosis and treatment at the highest national level (see Fig. 3).

## Migraine with and without aura

### Diagnosis

The two most common subtypes of migraine are migraine with aura and migraine without aura. Many patients have both types. Migraine without aura presents with attacks lasting between 4 and 72 h, and the most typical characteristics are unilateral headache of throbbing quality, moderate to severe intensity and with aggravation by routine physical activity [2]. The headache is typically accompanied by nausea, vomiting and phono and/or photophobia (see Tables 3 and 4).

**Table 3:** Classification of migraine without aura and typical aura with migraine headache [2]

1.1 [G43.0] Migraine without aura	
A. At least five attacks fulfilling criteria B-D.	
B. Headache attacks lasting 4–72 h (untreated or unsuccessfully treated)	
C. Headache at least two of the following four characteristics:	
1. unilateral location	
2. pulsating quality	
3. moderate or severe pain intensity	
4. aggravation by or causing avoidance of routine physical activity (e.g. walking or climbing stairs)	
D. During headache at least one of the following:	
1. nausea and/or vomiting	
2. photophobia and phonophobia	
Not better accounted for by another ICDH-3 diagnosis	
1.1 [G43.1] Migraine with aura	
A. At least two attacks fulfilling criteria B-C	
B. One or more of the following fully reversible aura symptoms:	
1. visual	
2. sensory	
3. speech and/or language	
4. motor	
5. brainstem	
6. retinal	
C. At least three of following six characteristics:	
1. at least one aura symptom spreads gradually over ≥5 min	
2. two or more aura symptoms occur in succession 3. each individual aura symptom lasts 5–60 min	
4. at least one aura symptom is unilateral	
5. at least one aura symptom is positive	
6. the aura is accompanied, or followed within 60 min, by headache	
D. Not better accounted for by another ICDH-3 diagnosis.

**Table 4:** Classification of chronic migraine [2]

1.3 [G43.3] Chronic Migraine	
A. Headache (migraine-like or tension-type-like) on ≥15 days for > 3 months, and fulfilling criteria B and C	
B. Occurring in a patient who has had at least five attacks fulfilling criteria B-D for Migraine without aura and/or criteria B-C for Migraine with aura	
C. On ≥8 days/month for > 3 months, fulfilling any of the following:	
1. criteria C and D for Migraine without aura	
2. criteria B and C for Migraine with aura	
3. believed by the patient to be migraine at onset and relieved by a triptan or ergot derivate	
D. Not better accounted for by another ICDH-3 diagnosis	

Patients are symptom-free between attacks [2]. Many patients suffer from both migraine and tension-type headaches. Table 5 lists some typical features of the two types of headaches. Approximately one-third of patients with migraine have migraine with aura [3]. The aura phase consists of lateralised reversible symptoms from the vision and tactile senses, such as flicker scotoma and sensory disturbances.

**Table 5:** Characteristics to distinguish between migraine and tension-type headache

	Migraine	Tension-type headache
Time pattern	Attacks lasting 4–72 h	Varies, from episodes lasting 30 min to a continuous headache
Headache characteristics	Frequently unilateral and pulsating. Aggrevation by physical activity	Frequently bilateral and pressing. Normally no aggravation by physical activity
Intensity	Tyically moderate to servere	Typically mild to moderate
Accompanying symptoms	Frequent nausea and/or vomiting, photophobia or phonophobia	None or only mild nausea, photophobia or phonophobia

Transient aphasia can also be seen. Typically, symptoms develop gradually over minutes, every aura symptom has a duration of 5–60 min, and different types of symptoms follow each other (see Table 3). If the aura includes motor weakness, it is classified as hemiplegic migraine. In migraine with aura, there is not necessarily a subsequent headache [2].

### Background

Migraine is very frequent with a lifetime prevalence of approx. 16% in Denmark [4]. Migraine occurs in all age groups, including children (see Section “Headaches in children”). There are more women than men with migraine, especially in migraine without aura, where the male/female ratio is 1: 3.3.

Recently, significant progress has been achieved in understanding the mechanisms behind migraine. Migraine is most likely a neurovascular disease in which genetic predisposition makes the brain of a migraine patient more susceptible to a variety of endogenous and exogenous trigger factors. Migraine aura is probably due to a transient slowly spreading decreased neuronal metabolism typically starting occipital, so-called cortical spreading depression [5]. Migraine headache is probably due to activation of nociceptors in the meninges and cerebral blood vessels and to secondary, increased pain sensitivity in the central nervous system [5].

### Clinical assessment and special assessment program

The use of a headache diary is essential to make the correct diagnosis (see Fig. 1, can be downloaded at dhos.dk), especially to distinguish between mild migraine attacks and tension-type headache and to rule out medication overuse headache. Comorbid diseases, e.g. hypertension, asthma, severe obesity and depression should also be diagnosed and managed. Comorbid conditions are crucial when choosing preventive medication. Migraine is a benign condition, but women with migraines with aura have an increased risk of stroke, even though the absolute risk is small (see Section “Hormones and migraines”). Typically, there is typically no need for paraclinical tests, see Section “Diagnosis and organization”.

### Non-pharmacological treatment

Non-pharmacological interventions are an important part of the treatment for some headache patients, although there is generally only sparse evidence for the effect of this type of intervention.

- Biofeedback therapy has a documented effect on migraine [6].
- Acupuncture in addition to symptomatic treatment has in a meta-analysis shown to reduce the number of headache days corresponding to preventive medical treatment, but there is only a small difference compared to placebo [7].
- There is consensus, but not clear scientific evidence, that physiotherapy should primarily focus on instruction in relaxation, correct working postures, posture correction and instruction in active home exercises [8].
- Physical exercise can be beneficial to reduce the headache duration, but not frequency or intensity [8].
- Behavioural therapy and cognitive therapy (stress and pain management) may be effective, but are only offered to a limited extent in Denmark.
- Information about the causes of migraine and the possibilities for treatment, thorough physical examination, as well as simply taking the patient seriously, can have a beneficial effect in some patients.
- Identify and reduce as much as possible, predisposing factors such as stress, depression or anxiety.
- Identify and eliminate triggers, e.g. irregular lifestyle (e.g. poor sleep patterns or irregular food intake). Intake of provocative food items such as red wine, dark chocolate and certain cheeses may in some patients have an effect, although there is no clear evidence for this.

### Pharmacological treatment

Medical treatment is divided into acute treatment and preventive treatment.

#### Acute treatment

##### General guidelines

- No definite difference has been demonstrated in the effect between simple analgesics (paracetamol, NSAIDs and acetylsalicylic acid) alone or in combination with antiemetics and triptans [9]. Simple analgesics, if necessary combined with antiemetics, are therefore the first choice [10]. Many of the patients who have insufficient effect of simple analgesics have good effect from triptans [10].
- Step-wise treatment is recommended in which each step comprises three treatments before proceeding to the next step. Hereby, the most effective and inexpensive treatment is achieved [10].
- The first step consists of simple analgesics and an antiemetic if needed.
- The second stage consists of triptans.
- Ergot alkaloids (ergotamine derivate) are rarely used due to the risk of serious side effects. These should only be used by specialists.
- Treatment should be initiated as early as possible. In migraine with aura, however, triptans should be taken only when the headache starts.
- Analgesics often has a better effect when combined with rest and, possibly, sleep. If the patient has difficulty calming down, benzodiazepine may be given e.g. 5 mg diazepam.
- Pay attention to medication overuse headaches (see Section “Medication overuse headaches”).

##### Simple analgesics and antiemetics

- The efficacy of paracetamol, acetylsalicylic acid and various NSAIDs for the treatment of migraine attacks has been demonstrated [10]. See Table 6 for suggested doses.
- For concomitant nausea, simple analgesics may be combined with antiemetics to treat the nausea, but there is no conclusive evidence that antiemetics improve the absorption of common analgesics [11]. Metoclopramide tablet 10 mg or tablet domperidone 10 mg can be used (the latter used especially for young people due to less risk of extrapyramidal side effects). See Table 6.
- Simple analgesics should be used for a maximum of 14 days per month to avoid medication overuse headache.

**Table 6:** Acute treatment of the migraine attack, first step: Simple analgesics and antiemetics with proven effects on the migraine attack and suggested initial doses [10]. These drugs can be used 2–3 times a day. Intake of combined analgesics should be limited to max. 9 days per month to avoid medication overuse headache. Tolfenamic acid 200 mg and diclofenac 50–100 mg can also be used

Analgesics	Initial Dose	Antiemetics	Initial Dose
Ibuprofen	400–600 mg	Metoclopramide	10 mg
Naproxen	500 mg	Domperidone	10 mg
Paracetamol	1.000 mg		
Aspirin/caffeine	500/50 mg		

##### Triptans

- There are clinically relevant differences between the seven oral triptans in terms of efficacy and side effects (Table 7). In addition, there can be significant differences in how well the individual patient responds to the different triptans, therefore the choice of triptan should be individualized.
- Patients who have no effect of one particular triptan may benefit from another triptan. Before the effect of any triptan is ruled out, the patient should, as a rule of thumb, have tried three different triptans, each during three attacks.
- There are significant price differences between the triptans.
- There is no evidence that the effect of orally disintegrating tablets or rapidly soluble tablets is any quicker than that of standard tablets. Nasal spray and subcutaneous injection act more rapidly than tablets.
- Triptans should be taken early in the attack (while the pain is mild) [12], but not during the aura phase, as they are not usually effective here [13]. It is important for the patient to be able to distinguish between migraine and tension headache to avoid overuse of triptans.
- A combination of triptan and NSAIDs may be more effective in some patients than each drug alone [14].
- Oral triptans may, in case of nausea/vomiting, be combined with the antiemetic metoclopramide [15] or domperidone; in some cases non-oral administration is an advantage (nasal spray or subcutaneous injection).
- Approximately 20–50% of patients experience recurrence of migraine within 48 h. An additional dose of triptan is usually effective in these cases. Recurrence of migraine can also be treated with NSAIDs.
- In the absence of effect of a triptan, repeating the treatment with triptan in the same attack is usually ineffective.
- Triptans should be used for a maximum of 9 days per month to avoid headache medication overuse.
- Common side effects are a sensation of pressure on the chest, nausea, distal paraesthesia and fatigue.
- Triptans have not been systematically tested in hemiplegic migraine or migraine with brainstem aura and is not recommended for use in these patients in some countries. However, studies in hemiplegic migraine and migraine with brainstem aura patients have not shown risk of using triptans [16, 17]. If these patients do not have previous clinical stroke, uncontrolled hypertension, ischemic heart disease or peripheral vascular disease, triptans can be used. Of note, experimental studies in humans have shown that sumatriptan do not constrict intracerebral vessels [18, 19].
- Triptans are generally contraindicated in patients with previous stroke. Some migraine patients can have accidental findings of white matter changes or infarct-like changes on brain imaging. If these patients do not present history of clinical stroke, uncontrolled hypertension, IHD or peripheral vascular disease, triptans can be used.
- Triptans are, among others, contraindicated in uncontrolled hypertension, ischemic heart disease, previous cerebral infarction and peripheral vascular diseases. Caution should be exercised when treating patients < 18 years and > 65 years. However, Sumatriptan 10 mg nasal spray is approved for adolescents aged 12–17 years. See www.promedicin.dk for a detailed list.

**Table 7:** Acute treatment of the migraine attack, second step: Triptans, which are available in Denmark (listed according to the date of marketing in Denmark). If there is an effect of the first dose of a triptan but the migraine recurs, an additional dose may be taken at least 2 h after the first dose. Therapeutic gain is the response to treatment of active substance minus the response to placebo

Triptan	Formulation	Therapeutic gain (pain freedom after 2 h)	Comments
Sumatriptan	Tablet 50 mg	17%	
	Tablet 100 mg	21%	
	Nasal spray 10 & 20 mg	21% (20 mg)	
	Subcutaneous injection 6 mg	44%	
Zolmitriptan	Tablet 2.5 mg Soluble tablet 2.5 mg	20%	Soluble tablets have same efficacy as tablets
Naratriptan	Tablet 2.5 mg	15%	Side effects equals placebo
Rizatriptan	Tablet & soluble tablets 5 and 10 mg	30%	5 mg if concomitant treatment with propranolol
Almotriptan	Tablet 12,5 mg	23%	Side effects equal placebo
Eletriptan	Tablet 40 mg	28%	80 mg suggested, if 40 mg does not work
Frovatriptan	Tablet 2.5 mg	8%	Probable slower onset of effect but longer lasting compared to sumatriptan

#### Preventive treatment

##### General guidelines

- Preventive treatment is offered to reduce the frequency or severity of attacks.
- Preventive treatment should be considered [10] if
  - The number of migraine days per month is four or higher.
  - attack medications have poor effect.
  - the patient’s quality of life is reduced considerably due to the migraine.
  - frequent or very long-lasting cases of aura occur.
- Thorough information to the patient about the purpose, side effects and realistic expectations about treatment effect is important.
- Preventive treatment is generally considered successful if the frequency or severity of migraine can be halved without the occurrence of bothersome side effects [10].
- Choose preventive medication based on scientific evidence for efficacy, side effect profile and competing disorders.
- Use slow titration to minimize side effects.
- Use a headache calendar to document the effect.
- The prevention treatment should attempted for a minimum of 2–3 months at full dose, before it may be finally assessed whether there is an effect (unless it is not tolerated due to side effects).
- In case of effect, the preventive drug should be discontinued every 6–12 months to ensure that there is still a need for and effect of the medication.
- Lack of effect of one type of preventive does not preclude effect of other types of prevention.
- There is no evidence of an effect when combining several forms of preventive.
- At ≥15 headache days per month, medication overuse should be ruled out.

##### Beta blockers

Beta-blockers with no autostimulating effect have a well-documented effect in migraines. Beta-blockers should normally be chosen as the first of first-line drugs. There is best evidence for propranolol and metoprolol [20]. Typical dosage for metoprolol is 50 mg × 1 for 1 week and then 100 mg × 1, increased to 150–200 mg per day in case of lack of effect. Typical dosage for propranolol is 40 mg × 2 increased at a weekly interval to a maximum of 120 mg × 2. There is often an effect at 120–160 mg daily. Once the dose has been determined, a retard drug can be used to increase compliance. There is some evidence for the effect of bisoprolol, timolol and atenolol [10]. Side effects include fatigue, dizziness, reduced physical ability and cool extremities. See Tables 8 and 9.

**Table 8:** Preventive medication against episodic migraine in a prioritized order and recommended doses for adult. Valproate should not be prescribed in women of childbearing age

Drug	Daily dose
Metoprolol/propranolol	50–200 mg/40–240 mg
Candesartancilexetil	16 (24–32) mg
Topiramate	25–100 (200) mg
Amitriptyline	10–100 mg
Flunarizin	5–10 mg
Valproate	500–1800 mg
Lisinopril	20 mg
Pitzotifen	1.5–3 mg
Riboflavin	400 mg
Magnesium	360 mg

**Table 9:** Preventive medication against chronic migraine with recommended doses

Drug	Daily dose
Beta-blockers:	
Metoprolol	50–200 mg
Propranolol	40–240 mg
Candesartancilexetil	16 (24–32) mg
Topiramate	50–100 (200) mg
Amitriptyline	10–100 mg
Botulinum type a toxin	155–195 units i.m. Every 12 weeks
CGRP-antibodies:	
Erenumab	70–140 mg s.c. every 4 weeks
Fremanezumab	225 mg s.c. every month or 675 mg s.c. every 3 months
Galcanezumab	Start dose 240 mg s.c. followed by 120 mg s.c. every month.
Eptinezumab	100–300 mg i.v. every 3 months

*s.c.* subcutaneous, *i.m.* intramuscular, *iv.* intravenous

##### Angiotensin II receptor antagonist

Candesartancilexetil has few side effects and an effect comparable to beta-blockers [21]. The typical dosage for candesartancilexetil is 8 mg × 1 for 1 week and then 16 mg × 1, possibly increasing to 24–32 mg × 1. See Tables 8 and 9.

##### Anti-epileptics

Topiramate and valproate have well-documented effects that are comparable to beta-blockers, but are generally associated with more side effects. Typical dosage for topiramate is 25 mg × 1 increasing by 25 mg at 14-day intervals to 100 mg daily in two divided doses. Later, dose adjustment can be made to 50–200 mg daily in two divided doses. Side effects include paraesthesia, sedation, dizziness, weight loss, kidney stones and cognitive side effects. Typical dosage for valproate is 1000 mg × 1. Later dose adjustment to 500–1800 mg × 1 can be made [10]. Side effects include dyspepsia, hand tremor, weight gain, liver affection, thrombocytopenia and fetal malformations. Valproate should not be prescribed in women of childbearing age. See Tables 8 and 9.

##### Antidepressants

Amitriptyline is particularly suitable if the patient also suffers from frequent tension-type headache or chronic migraine [10]. Typical dosage is 10 mg × 1 increasing by 10 mg at one-week intervals to 10–100 mg daily. The full dose is given 1–2 h before bedtime. Typical dose where the best balance between effect and side effects is 30–70 mg daily. For more details see chapter 4.

##### Calcium channel blockers

Flunarizine has a comparable effect to beta-blockers, but is generally associated with more side effects. Normal dosage is 10 mg × 1, but elderly patients should only receive half the dose [10]. Side effects include drowsiness, fatigue, weight increase, depression and unmasking of latent Parkinson disease. See Table 9.

##### Botulinum type a toxin

Botulinum type A toxin (Botox) is in Denmark so far only approved for the preventive treatment of chronic migraine (headache ≥15 days per month, of which at least 8 days with migraine) in patients who have shown insufficient response or intolerance to other migraine preventive drugs. Medication overuse should be attempted to be treated before initiating botulinum type A toxin [22]. Treatment is a headache specialist task. See Table 9.

##### CGRP antibodies

Four antibodies have been developed that bind to calcitonin gene-related peptide (CGRP) or its receptor, which has proven efficacy as a preventive treatment for episodic and chronic migraine [23]. The drugs are given as a subcutaneous injection or intravenous infusion at 4 weeks, 1 month or three-month interval and is a headache specialist task. Erenumab blocks the CGRP receptor. The other three antibodies (fremanezumab, galcanezumab and eptinezumab) bind to the CGRP ligand. In Denmark, erenumab and fremanezumab are currently recommended as possible preventive treatment for patients with chronic migraine who have experienced treatment failure in previous preventive treatments with at least one anti-hypertensive and one anti-epileptic. Eptinezumab has received FDA approval and is awaiting EMA approval. Any medication over use headache should be attempted to be treated before initiating treatment. In Denmark, the right to prescribe CGRP antibodies is limited to specialists in neurology who are employed in a hospital. CGRP antibodies are dispensed from hospital. The Danish Medicines Agency has established national criteria for treatment with CGRP antibodies. See the Danish Medicines Agency’s website. It is expected that there will be a significant development within the CGRP area in the coming years. See Table 9.

##### Second- and third-line drugs

There are several drugs that have less evidence of efficacy or more side effects than the above-mentioned first-choice drugs.

Naproxen dosage is 500 mg × 2 and can also be used for shorter periods in the treatment of menstrual-related migraines [24] (see section on migraines and hormones). Pizotifen is dosed as 0.5 mg every 3 days to 1.5 mg nocte, possibly increasing to 1 mg × 3. The side effects include weight gain and fatigue.

## Tension-type headache

### Diagnosis

Tension-type headache is predominantly characterized by a bilateral, pressing pain of mild to moderate intensity. The headache is only to a lesser extent or not at all associated with the typical migraine characteristics such as aggravation by physical activity, vomiting or severe nausea and hypersensitivity to light and sounds. There are three types of tension headaches that differ in the number of days per month with headaches; 1) sporadic episodic tension-type headache, 2) frequent episodic tension-type headache and 3) chronic tension-type headache. The diagnostic criteria for tension-type headache is listed in Table 10.

**Table 10:** Classification of tension-type headache [2]

2.1 [G44.2] Infrequent episodic tension-type headache	
A. At least 10 episodes of headache occurring on < 1 day/month on average (< 12 days/year) and fulfilling criteria B-D	
B. Lasting from 30 min to 7 days	
C. At least two of the following four characteristics:	
1. bilateral location	
2. pressing or tightening (non-pulsating) quality	
3. mild or moderate intensity	
4. not aggravated by routine physical activity such as walking or climbing stairs	
D. Both of the following:	
1. no nausea or vomiting	
2. no more than one of photophobia or phonophobia	
E. Not better accounted for by another ICHD-3 diagnosis.	
2.2 [G44.2] Frequent episodic tension-type headache	
As 2.1 apart from:	
A. A minimum of 10 episodes of headache occurring on ≥1 day/month but < 15 days/month on average (≥12 and < 180 days/year) and fulfilling criteria B-D	
2.3 [G44.2] Chronic tension-type headache	
As 2.1 apart from:	
Headache occurring on ≥15 days/month on average for > 3 months (≥180 days/year), fulfilling criteria B-D	
A. A minimum of 10 episodes of headache occurring on ≥1 day/month but < 15 days/month on average (≥12 and < 180 days/year) and fulfilling criteria B-D	
B. Lasting hours to days, or unremitting	
C. Both of the following:	
1. no more than one of photophobia, phonophobia or mild nausea	
2. neither moderate or severe nausea nor vomiting	

### Background

Episodic tension-type headache, which occurs no more than a few times a month, rarely causes concern. The headache will often be the body’s warning signal of inexpedient strain, e.g. due to stress or unphysiological work postures. Frequent episodic and chronic tension-type headaches, on the other hand, can be very bothersome and can significantly reduce quality of life [25].

The mechanisms behind tension-type headaches are not fully elucidated, but in the episodic form, referred pain from pericranial musculoskeletal tissues and stress probably play an important role. In patients with frequent episodic and chronic tension-type headaches, the central nervous system has been shown to be hypersensitive to pain stimuli. This may be due to insufficient inhibition of incoming painful stimuli from muscles or be a consequence of frequent painful inputs from the pericranial musculoskeletal tissues (central sensitization) [26].

### Clinical assessment and special assessment program

The headache diary is essential to make the correct diagnosis, especially to distinguish between tension-type headache and mild migraine attacks and to exclude medication overuse headache (see Fig. 1, can be downloaded at dhos.dk). Physical examination is important, partly to clarify any musculoskeletal causes of the headache, and partly to rule out more serious secondary causes. Such reassurance can have a beneficial effect in patients who have been worried that they might have a brain tumor. The examination should include palpation of the pericranial muscles for tenderness to assess the degree of musculoskeletal tension as well as assessment of the chewing apparatus for bite dysfunction. Comorbid diseases, especially depression, must also be diagnosed and treated. Many patients want to have an imaging examination of the neck, but this is only indicated by specific suspicion of cervical pathology. Regarding the need for paraclinical examinations in general, see Section “Diagnosis and organization”.

### Non-pharmacological treatment (see Table 11)

- Treatment of tension-type headaches is based primarily on non-pharmacological interventions. There is little or no scientific evidence for this, so the following recommendations are based on “expert opinion” [27].
- Identify and eliminate, to the extent possible, triggers, e.g. stress or unphysiological work postures. Physical activity may be beneficial.
- Provide information about the causes of tension-type headaches. It may be explained that each headache episode can be due to muscle tension or stress, while in chronic headache there may be a disturbance in the pain-regulating centres of the brain.
- Physiotherapy should primarily be aimed at instruction in correct working postures, posture correction and instruction in active home exercises aimed at reducing musculoskeletal tensions [27]. Passive physiotherapy has no lasting effect and is not recommended.
- Behavioural therapy and cognitive therapy (stress and pain management) are typically handled by psychologists. The treatment involves instruction in relaxation, biofeedback (electromyography (EMG) and temperature) and cognitive techniques (including restructuring of negative thoughts). The focus is on managing pain and stress [27]. This type of treatment is currently only offered in a few places in Denmark. Biofeedback therapy has a documented effect on tension headaches [6].
- Acupuncture has a documented effect [28]. However, only a minimal effect following active treatment was found compared to sham treatment.
- Manipulation of the cervical spine and blockage of the greater occipital nerve have not had an effect in the few controlled studies performed.

**Table:** Table 11

Non-pharmacological treatment of tension-type headache	
• Physical and neurological examination and reassurance.	
• Rule out other underlying disorder e.g. depression or oromandibular dysfunction.	
• Rule out overuse of analgesics	
• Inform the patient about pain mechanisms	
• Minimize, as possible, provoking factors, e.g. stress or non-physiological work posture	
• Physiotherapy (daily exercises and posture correction)	
• Biofeedback	
• Stress- og pain management	
• Acupuncture	

### Pharmacological treatment (see Table 12)

#### Attack treatment

There is a well-documented effect of weak analgesics in the individual episodes of tension-type headaches, while the effect is often limited in chronic tension-type headache [29]. The following treatments are recommended [27].

- Ibuprofen 400 mg (200–600 mg)
- Aspirin 1000 mg (500–1000 mg)
- Naproxen 500 mg (250–500 mg)
- Paracetamol 1000 mg

**Table:** Table 12

Pharmacological treatment of tension-type headache	
• Treatment of the individual episode (NSAID, paracetamol).	
• Avoid overuse of analgesics.	
• Avoid opioids.	
• Prophylactic treatment should be considered for chronic tension-type headache when there is insufficient effect of non-pharmacological treatment.	
• Amitriptyline, mirtazapine and venlafaxine may have preventive effects.	
• Remember to inform that antidepressants are given to increase the concentration of pain-inhibitory neurotransmitters in the central nervous system and not to treat depression.	
• Use a headache calendar to monitor the treatment effect	
• Prophylactic medication should be discontinued after 6–12 months to see if there is still a need for the medication.	

Controlled studies suggest that NSAIDs (including aspirin) are more effective than paracetamol [27]. Diclofenac has a higher risk of cardiac side effects than ibuprofen and naproxen and is therefore not recommended. The choice of weak analgesics must be made based on effect and side effects for the individual patient. It is extremely important to assess whether there is an effect at all or whether analgesics are taken automatically (“to at least do something”) and to set limits to avoid overconsumption. Simple analgesics should be used for a maximum of 14 days per month. Codeine and various combination products should be used for a maximum of 9 days per month to prevent headache overdose (Section “Headache medication”). Morphine should be avoided.

#### Preventive treatment

Preventive treatment may be indicated in patients with chronic tension-type headache, if there is insufficient effect of non-pharmacological treatment and when medication overuse headache is excluded [27]. Several placebo-controlled studies have shown an effect of the tricyclic antidepressant amitriptyline [29], which is first choice for preventive treatment of chronic tension-type headache. The effect is independent of any depression present. The newer serotonergic and noradrenergic antidepressants mirtazapine (30 mg/day) and venlafaxine (150 mg/day) have both been reported to be effective in single studies [29]. They can be used if amitriptyline has no effect or in cases of concurrent depression. Mirtazapine had comparable effects to amitriptyline. There is no documented effect of treatment with selective serotonin reuptake inhibitors (SSRIs), muscle relaxants or botulinum toxin [29].

General guidelines:

- Inform thoroughly about the mechanisms of action (especially that antidepressants are not given on the indication depression) and side effects.
- Slow escalation to minimize side effects.
- Use a sufficiently high dose.
- Monitor effect using a headache calendar (see Fig. 2, can be downloaded at dhos.dk or downloaded as an app for smartphone).
- Asses the effect after 1–2 months on the final dose.
- Attempt discontinuation every 6–12 month.

1. Amitriptyline tablets, 10–75 mg nocte. Effect: 30% reduction of headache compared to placebo.
  ECG should be checked before starting treatment and again at doses above 40 mg / day.
  10 mg × 1, increasing by 10 mg per week until an effect is achieved or significant side effects occur.
  The maintenance dose where the best balance between effect and side effects is typically is 30–75 mg.
  The full dose is given 1–2 h before bedtime to improve sleep and minimize fatigue the next day.
  Typical side effects include dry mouth, fatigue, dizziness and weight gain.
  Nortriptyline can be considered as an alternative used in the same way and with the same doses as amitriptyline. It probably improves sleep less than amitriptyline but also causes less sedation [30].
2. Mirtazapine tablets, 30 mg nocte. Effect: 30% reduction of headache compared to placebo.
  15 mg × 1, increasing to 30 mg × 1 after one week.
  Administered approximately one hour before bedtime.
  Typical side effects include fatigue, weight gain and dizziness.
3. Venlafaxine 150 mg tablets. Effect: 20% reduction of headaches.
  75 mg × 1, increasing to 150 mg × 1 after one week.
  Administered approximately one hour before bedtime.
  Typical side effects include fatigue, abdominal pain, nausea and dizziness.

### Summary

Tension-type headaches is the most common primary form of headache and is a substantial health problem in its frequent episodic or chronic form. In episodic tension-type headache, reported pain from pericranial musculoskeletal tissues as well as stress are likely to play an important role, while altered central pain modulation is involved in the chronic form. Correct diagnosis is important, especially differentiation between episodic tension-type headache and migraine. Comorbid factors, e.g. depression, as well as secondary headaches, e.g. medication overuse headache, should be ruled out. The treatment is based primarily on non-pharmacological measures such as information, minimizing triggers, physiotherapy with posture correction and instruction in active exercises as well as stress and pain management. The individual episode can be treated with weak analgesics. In patients with chronic tension-type headache, analgesics rarely have an effect, so preventive treatment with amitriptyline, mirtazapine or venlafaxine may be indicated.

## Cluster headache

### Diagnosis

The pain of cluster headache is often described as one of the most severe and intense types of pain known to humankind. The attacks are unilateral, stabbing or drilling in character, and are periorbitally-, retroorbitally- or temporally located. The attacks are usually side-locked. Each attack typically lasts 15–180 min and may occur up to 8 times a day. The pain is accompanied by ipsilateral autonomic symptoms like tearing, rhinorrhoea, ptosis, miosis and eyelid oedema due to parasympathetic hyperactivity and sympathetic hypoactivity. In contrast to patients with migraine, a patient with cluster headache is restless and agitated during the attacks [2, 31]. Migraine-like features such as nausea, photo- and phonophobia can be seen in cluster headache attacks as well and patients may experience mild-moderate pain attacks between the severe attacks [32]. Attacks often show a striking rhythmicity as they often occur at the same time of day and year, and most patients experience that they are awaken by the attacks from sleep [33].

Cluster headache is divided into 2 types: An episodic type seen in 80–90% of patients, where the attacks occur in bouts lasting 4–12 weeks separated by attack-free periods of varying length (weeks-years); and a chronic type seen in 10–20% of patients, with bouts lasting longer than 9 months per year. See Table 13.

**Table:** Table 13

3.1 [G44.0/N90] Cluster headache [2]	
A. At least five attacks fulfilling criteria B-D	
B. Severe or very severe unilateral orbital, supraorbital and/or temporal pain lasting 15–180 min (when untreated)^1^	
C. Either or both of the following:	
1. at least one of the following symptoms or signs, ipsilateral to the headache:	
- Conjunctival injection and/or lacrimation	
- Nasal congestion and/or rhinorrhoea	
- Eyelid oedema	
- Forehead and facial sweating	
- miosis and/or ptosis	
2. a sense of restlessness or agitation	
D. Occurring with a frequency between one every other day and 8 per day^2^	
E. Not better accounted for by another ICHD-3 diagnosis	
3.1. [G43.01/N89] Episodic cluster headache	
A. Attacks fulfilling criteria 3.1 and occurring in bouts (cluster periods)	
B. At least two bouts lasting from 7 days to 1 year (when untreated) and separated by pain-free remission periods of ≥3 months	
3.1.2 [G43.02/N89] Chronic cluster headache	
A. Attacks fulfilling criteria 3.1 and occurring in bouts (cluster periods)	
B. Occurring without a remission period, or with remission lasting < 3 months, for at least 1 year	

1: During part, but less than half, of the active time-course, attacks may be less severe and/or shorter or longer duration

2: During part, but less than half, of the active time-course, attacks may be less frequent

### Background

The age of onset is typically 20–40 years but young children may be affected too. The prevalence is 80–100 per 100.000 people in the general population [34]. Men are affected 2–4 times more often than women [2, 31]. The disease is unpredictable and attacks may follow one pattern for years but may suddenly change. Overall, it often becomes less severe with age.

The mechanisms behind cluster headache are far from established. Due to the striking rhythmicity of attacks, it is believed that cluster headache originates from the hypothalamus, which is involved in the regulation of pain, sleep and circadian rhythm [35]. Imaging studies support this theory [36, 37]. In addition, it is known that the trigemino-autonomic reflex is activated during attacks, which explains the pain distribution and the presence of autonomic symptoms [38].

### Clinical evaluation

If patients suffer from episodic cluster headache and have a normal neurological examination, neuroimaging is unnecessary. In chronic cluster headache, in patients with an atypical presentation, onset after 40 years of age or in treatment refractory cluster headache, a cerebral MR scan should be performed to exclude tumours, midline malformations, pathology in the cavernous sinus, pituitary gland and hypothalamus [39]. The differential diagnoses include other trigeminal autonomic cephalalgias (TACs), which are often distinguished according to attack duration and frequency (Table 14). However, the most common misdiagnoses are migraine, tension-type headache and sinusitis [31]. A headache diary is often very useful.

**Table:** Table 14

	Cluster headache	Paroxysmal hemicrania	SUNCT
Epidemiology			
Gender ratio (M:F)	2–4:1	1:2–3	8–12:1
Prevalence	0,9%	0,02%	Very rare
Typical age of onset	20–40 years of age	20–40 years of age	20–50 years of age
Pain			
Character	Drilling/stabbing/squeezing	Drilling	Stabbing
Intensity	Very severe	Severe	Severe
Localization	Periorbital	Orbital, temporal	Orbital, temporal
Attack duration	15–180 min	2–30 min	1–600 s.
Attack frequency	1–8 per day	1–40 per day	3–200 per day
Autonomic symptoms	Yes	Yes	Yes
Effect of indomethacin	No	Yes	No
Attack treatment	Oxygen 12–15 l/min Inj. Sumatriptan Nasal spray sumatriptan	None	None
Prophylactic treatment	Verapamil, prednisone	Indomethacin	Lamotrigine, Topiramate, Gabapentin

### Non-pharmacological treatment

Generally, non-pharmacological treatment has not been shown to have an effect in cluster headache [39].

### Pharmacological treatment (Table 15)

General recommendations:

- If cluster headache is suspected, patients should be referred sub-acutely to a private neurologist or a department of neurology to facilitate correct diagnosis and thereby correct treatment.
- Patients should immediately receive acute treatment for the attacks and preventive treatment aiming to reduce attack frequency and pain intensity.
- The dosage of the preventive treatment should be increased as fast as possible.
- The dosage of the preventive treatment should be gradually reduced if patients are attack-free for 14 days (please be aware that patients may experience milder attacks and/or autonomic symptoms indicating that the bout is still active) or when patients sense that the bout has ended.

**Table 15:** Pharmacological treatment of cluster headache

Type	Dose
Attack treatment:	
Inhalation of 100% oxygen	12–15 l/min via O_2_ptimask [36] w. 3 l reservoir or DVO mask
Inj. sumatriptan	6 mg
Sumatriptan nasal spray	20 mg
Preventive treatment	
Tablet verapamil retard	Initially 100 mg × 2 for 3 days, hereafter 200 mg × 2. Possible further increase to 400–600 mg daily. Rarely up to 1000 mg
Transitional treatment	
Occipital nerve block (GON) / tablet prednisone	Please refer to text

*DVO* demand valve oxygen

#### Acute treatment

##### Oxygen inhalation

Oxygen is a safe treatment with no side effects or contraindications. First line treatment is inhalation of 100% oxygen through a non-rebreather mask with a flow of 12–15 l per minute. Within 30 min this is effective in 60% of the patients [40]. A sumatriptan injection may often be used in addition [40]. The inhalation of oxygen should be performed in upright position and as early in the attack as possible. Studies have shown that mask type is important for the treatment to be efficient [41]. Patients should start with an O_2_ptimask with 3 l reservoir and if the effect is insufficient, they should try an oxygen demand valve mask before the effect of oxygen can be excluded. Transportable oxygen equipment is often supplied by private companies on prescription from a department of neurology. Such equipment is normally supplied on the day it is ordered. As this service is relatively costly, the patient should only have the equipment for periods with cluster headache. Patients should contact the oxygen company with an option of retrieving the oxygen cylinder once their cluster period ends. There may be regional differences in the rules concerning when oxygen cylinders should be returned.

##### Triptans

Subcutaneous injection of sumatriptan 6 mg relieves pain completely in about 75% within 15 min and is therefore considered 1st line treatment if oxygen is insufficient or if the patients need a treatment more easily handled when not at home [42]. Sumatriptan nasal spray 10 mg or 20 mg can also be effective, but pain is relieved more slowly and the patients should be instructed in correct administration (should be used on the attack-free side, with the head tilted forward, the tip pointed against the ear and the patient must not breathe inwards when the medication is released) [42]. The price of the nasal spray is lower than for the injections. The effect of oral triptans is too slow and they are not recommended, as doubt may occur to whether the attack ended by itself or whether it was the effect of the triptan. Patients with cluster headache are also at risk of developing medication overuse headache, but a daily use over a shorter period should be allowed.

##### Other acute medications

There is no treatment effect of neither simple analgesics nor opioids in cluster headache.

#### Preventive treatment

##### Verapamil

Verapamil is the first-line preventive treatment of cluster headache [42]. The start dose is typically 100 mg two times a day for 3 days, hereafter increase to 200 mg two times a day. From here, the dose can be increased by 100 mg every 7th day up to 600 mg daily.

The typical therapeutic dosage range is 400 to 600 mg daily. At times, it may be necessary to increase verapamil up to 1000 mg daily. Electrocardiogram must be performed before initiation (and before initiation in each bout) and must be repeated when increasing above 400 mg, 600 mg and 800 mg daily, in order to detect an atrioventricular block. Typical side effects are constipation, nausea, oedema, fatigue, hypotension, bradycardia and eczema. Initiation must be very cautious if the patients suffer from heart failure, atrioventricular block and when used in combination with beta-blockers.

##### Glucocorticoids

Glucocorticoids can be used in transition phases to achieve a quick relief before the effect of other preventive treatment is sufficient or if patients have a very short bout [42].

Prednisone can be administered in two ways:

- A greater occipital nerve block (GON-block) with 2 ml of betamethasone and 0.5 ml of lidocaine 20 mg/ml injected in the neck. The block should be injected halfway between protuberantia occipitalis externa and processus mastoideus, where the greater occipital nerve can be palpated. It is not complicated to perform the block and it is not necessarily a specialist task. If efficient, the effect typically lasts three to 4 weeks. Three months must pass between blocks to avoid tissue necrosis and alopecia.
- Prednisone tablets 75 mg once a day for 5 days hereafter reducing the dose with 12.5 mg a day, is also a very efficient treatment.

##### Lithium carbonate

Lithium carbonate may occasionally be used in chronic cluster headache or in episodic cluster headache in patients with very long bouts if the patients do not have effect of verapamil. The treatment requires close follow up and is usually accompanied by many side effects. Treatment with lithium carbonate should be administered by a neurologist.

##### Other treatment possibilities

Melatonin 6–9 mg before bedtime may have effect in some patients, improving night sleep and reducing the attack burden [42]. Some reports also show that topiramate 100–200 mg daily may have an effect [42]. In some treatment refractory patients, neuromodulation should be considered. Neurostimulation of the shenopalatine ganglion has shown positive long-term results in chronic medically refractory patients with 61% having acute and/or preventive effect [43]. Unfortunately, the treatment is currently unavailable. There is an unmet need for effective treatment of medically refractory patients. This may be covered with the CGRP antibodies and other future drugs targeting neuropeptides in the trigemino-autonomic reflex. In episodic cluster headache, Galcanezumab, one of the CGRP antibodies, has been shown to be effective in reducing attack frequency. So far, studies in chronic cluster headache have been terminated, due to difficulties in fulfilling the primary endpoints [44].

### Summary

Cluster headache is a highly disabling disease with unilateral, extremely severe pain attacks in the periorbital area, accompanied by ipsilateral autonomic symptoms and/or restlessness. Both acute and preventive treatment must be initiated quickly to relieve the burden.

## Medication overuse headache

### Diagnosis

Medication overuse headache (MOH) is a chronic headache occurring at least 15 days a month in patients with pre-existing headache. MOH is secondary to overuse of short-term medication, i.e. analgesics and migraine medication. An overuse is defined as a long-term (at least 3 months) use of simple analgesics (paracetamol and NSAIDs) ≥ 15 days/month, or intake of triptans, combination-analgesics, ergotamines, opioids, or any combination of the mentioned drug-classes ≥10 days/month [2]. The most recent diagnostic criteria are listed in Table 16. Importantly, patients fulfilling the criteria for MOH should be given the MOH-diagnosis AND the diagnosis of the pre-existing headache, e.g. chronic migraine AND MOH.

**Table 16:** Diagnostic criteria for medication overuse headache [2]

A. Headache occurring on ≥15 days/month in a patient with a pre-existing headache disorder	
B. Regular overuse for > 3 months of one or more drugs that can be taken for acute and/or symptomatic treatment of headache:	
1. Simple analgesics (paracetamol or NSAIDs) for ≥15 days/month	
2. Ergotamines, triptans, opioids, combination analgesics or any combination of the above mentioned ≥10 days/month	
C. Not better accounted for by another ICHD-3 diagnosis.	

Clinically, MOH presents with increasing headache frequency over months to years, often with more migraine attacks, longer duration, higher intensity and changes in the headache pattern from migraine-like headache to tension-type headache features. Patients often complain about that the short-term medication is ineffective. The same is true for pharmacological and non-pharmacological treatments.

The headache frequency and intensity will for most patients decrease when the medication overuse is withdrawn (withdrawal therapy), and the headache will return to the original pattern. Moreover, the headache will respond to short-term and preventive headache medication, as well as non-pharmacological treatment [45–47].

Noteworthy, patients with pre-existing headache are also at risk for developing MOH, when using analgesics for other conditions, e.g. low back pain [48].

### Background

The MOH prevalence in Denmark is approximately 2% of the adult population, and is seen more and more among children and adolescents [49]. The male:female ratio is 1:1.9 and MOH is most common in the ages of 30–50 years [49]. The most common pre-existing headache diagnoses are tension-type headache (10–43%), migraine (20–65%) or a combination of tension-type headache and migraine (30–49%). Studies based on populations from the primary sector reported a higher percentage of MOH-patients with pre-existing tension-type headache, compared to studies based on populations from the secondary or tertiary sector [45–47, 50] .Only a minority of patients with MOH has other pre-existing headache diagnoses (1–10%). As mentioned above, MOH can be developed based on all kinds of analgesics and acute migraine medication [2]. However, patients with pre-existing headache using combination-analgesics containing codeine, caffeine and/or barbiturates are at higher risk of MOH compared to those using simple analgesics [51]. Many pieces of the puzzle are still missing in understanding the pathophysiology in MOH. However, upregulation of central serotonergic and dopaminergic transmitter systems may be involved, and it is well-known that patients with MOH exhibit symptoms of central sensitization that is normalized after withdrawal therapy [52].

### Clinical assessment

A detailed patient interview, normal general physical and normal neurological examination are necessary before the MOH-diagnosis can be established. Moreover, the patients should fill out a diagnostic headache diary, including information about short-term medication, for at least 4 weeks before attending the physician (see Fig. 1). In addition, other secondary reasons causing headache must be ruled out. Even though a patient in principle fulfils the MOH-criteria already after 3 months, it is reasonable thinking about differential headache diagnoses if the headache has developed and escalated over short term. Most patients with MOH experience the worsening of headache over a period of months to years.

### Treatment of MOH

#### Non-pharmacological treatment

MOH is treated by withdrawal therapy (stop of the overuse of short-term medication) [53], either by a complete stop of all short-term medication for a 2 months period, or by a reduced intake of short-term medication to maximum 2 days a week in average. A complete stop has proven most effective in treating MOH [47]. Before starting withdrawal, it is crucial that the patient is informed properly about MOH, the rationale for withdrawal therapy and treatment of potential temporary withdrawal symptoms (Table 17). Information should include, that patients can experience the withdrawal symptoms in the first couple of weeks during the withdrawal period, including rebound headache with migraine-like features, nausea, vomiting, sleep disturbances, restlessness, anxiety, hypotension and tachycardia. It is recommended to rest and being well hydrated during the withdrawal period. Patients will often experience that the headache attacks become less intensive, shorter lasting and can be managed without short-term medication. Withdrawal symptoms typically lasts 2–10 days, depending on the type of medication overused (e.g. 2–3 days for triptans and 9–10 days for NSAIDs on average) [54]. Support from relatives, employer and health care professionals during withdrawal therapy is essential. If possible, for the patients, 2–3 weeks of planned sick leave is recommended.

**Table 17:** Non-pharmacological treatment of medication overuse headache

Elements of treatment	
• Complete stop of all kinds of short-term medication, including analgesics and acute migraine medication for 2 months. Alternatively, reduced intake of short-term medication to maximum 2 days a week, but this approach works less effectively.	
• Detailed information to patients, relatives and health care professionals.	
• Support, information and treatment of withdrawal symptoms.	
• In-patient care at a neurology department when substantial co-morbidities or high risk of severe withdrawal symptoms.	
• Recommendation of 2–3 weeks of sick leave.	
• Close follow-up during the first year after withdrawal.	

Withdrawal therapy often reverts the chronic headache pattern to an episodic pattern, and when the withdrawal symptoms wear off, patients experience a spontaneous improvement of their headache over weeks to months. Moreover, many patients also feel an improvement in their general health condition and well-being due to less short-term medication intake.

It has been shown that even a brief intervention in primary care can lead to a 50% reduction in MOH [55]. Referral of the patients to specialists and possible in-patient care at neurology departments should be considered in case of excessive medication overuse, overuse of opioids or barbiturates, severe co-morbidities (co-existing chronic pain conditions or psychiatric disorders) or previous unsuccessful withdrawal therapy (Table 17).

#### Pharmacological treatment during withdrawal therapy

Rescue medication can be useful in the first 1–3 weeks of withdrawal and should be discontinued hereafter (see Table 18). The risk of relapse is highest within the first year after withdrawal [56]. Relapse can be prevented by close follow-up of patients by health care professionals. Patients should be informed about the limits for use of short-term medication. A headache calendar is useful to count days with headache and use of medication (see Fig. 2).

**Table 18:** Pharmacological treatment for medication overuse headache

Rescue medication in week 1–3 of the withdrawal may be needed:	
• Tablet levomepromazine 12.5–25 mg as needed max. 75 mg/day, or tablet promethazine 25 mg as needed max. 75 mg/day.	
• Tablet metoclopramide 10 mg or tablet domperidone 10 mg against nausea as needed max. 30 mg/day.	
• In case of opioid- or barbiturate overuse: Tablet methadone, e.g. 20 mg, dosage decreased over next 4 days (only in-patient care).	
• Early start of preventive headache medication simultaneously with start of withdrawal should be considered	
After 2 months of withdrawal	
• If not started earlier, start of preventive headache medication should be started.	
• Detailed information to the patient about correct use of short-term medication, preventive medication, and headache calendar to prevent relapse.	
• Treatments that previously were ineffective due to medication overuse, may become effective after withdrawal.	

#### Preventive medication for MOH

For decades, Danish guidelines have recommended to postpone start of preventive headache medication to the end of 2 months withdrawal therapy for two reasons: 1) The headache pattern becomes clearer during withdrawal, and a correct diagnosis would help find the best treatment option for preventive medication; 2) It seemed that some patients did not need preventives after withdrawal.

However, a recently Danish study has reported that early start of preventive medication simultaneously with start of withdrawal therapy is more effective in treating MOH, compared to a strategy where preventive treatment is postponed to after withdrawal [57]. This may be due to fewer patients starting preventive medication when assigned to the last-mentioned strategy. Therefore, it is now recommended to start preventive medication together with start of withdrawal. In some cases, e.g. with very unclear pre-existing headache diagnosis, the start of preventive medication can be delayed with 2 months. The choice of preventive medication depends on the pre-existing headache (see specific sections).

### Summary

MOH is a chronic and secondary headache caused by overuse of short-term medication. Out-patient withdrawal therapy is feasible for most patients when detailed information is given, and they are well-prepared [50]. Nevertheless, the course can still be tough for the patient, who will require high level of support. In far most cases, withdrawal and preventive medication will lead to reduction in headache frequency and in intensity, even in patients with treatment resistant headache [46].

In principle, MOH should be prevented via information to patient with pre-existing headache and a restrictive approach to prescription of short-term medication. 

## Secondary types of headache

### Diagnostic criteria

Secondary headache is defined as a headache that occurs in a close temporal relationship to another disease that may cause headache [2]. This also applies if the emerging headache is clinically similar to a primary headache (e.g. migraine or tension-type headache). If an existing primary headache is significantly exacerbated (typically a doubling in attack frequency or pain intensity) or chronically in a close temporal context to a disease known to cause headache, both the primary and secondary headache diagnosis should be given.

### Background

Headache can appear as the first symptom of a serious life-threatening illness. Although the severe conditions represent far less than 1% of all types of headaches, a new onset, severe headache demands for attention and thorough clinical investigation. In the general population, approximately 2% a have secondary headache, which most often is medication overuse headache or post-traumatic headache. The time course of headache development is a very important element in the diagnosis. A “popping” or “snapping” painful sensation in the head with maximum pain intensity within a few seconds should always give suspicion of subarachnoid haemorrhage, while a subacute onset, progressive headache after a head trauma may be the first symptoms of increased intracranial pressure and a possible epidural hematoma that untreated can develop into a life-threatening condition within a few hours. Subacute headaches that develop over 1–2 days and are accompanied by fever, malaise and any seizures can e.g. be signs of meningoencephalitis, cerebral abscess or a cerebral sinus vein thrombosis. In contrast, a gradual onset headache developed over weeks to months, accompanied by possible epileptic seizures, personality change, speech disorders, and / or hemiparesis may represent a space-filling cerebral tumour or a chronic subdural hematoma.

### Special investigation program

The medical history and the neurological examination are fundamental elements for identifying a secondary headache. A thorough neurological examination, including ophthalmoscopy and measurement of temperature and blood pressure, is important. There is a secondary cause of the headache in approximately 3% of patients with red flags examined in the emergency department [58]. There may be an indication for CT or MRI scan of the cerebrum, MRI or CT angiography -venography, and lumbar puncture with pressure measurement as well as analysis of cerebrospinal fluid for cells, glucose and protein.

### Clinical assessment

Warning signals identified from the history or the physical examination that warrant further examination [59]:

- New onset headache
- Thunderclap headache (sudden onset of severe headache)
- Sudden headache occurring during strenuous physical or sexual activity
- Headache with atypical aura (lasts over 1 h or includes motor outcomes)
- Headache with aura developed while using birth control pills
- New onset of headache in a patient with cancer or HIV infection
- Headache accompanied by fever
- Headache accompanied by focal neurological signs except transient attributed to migraine aura
- Progressive headache over weeks
- New onset headache in patients under 10 years of age and over 40 years of age
- Headache that is position dependent

Refer to the general textbooks for a more detailed description of the many causes of secondary headaches, but the following forms can be highlighted:

#### Chronic post-traumatic headache

Debut / worsening of pre-existing headache within 7 days after the head trauma and continued headache after 3 months.

- In the vast majority of cases, the headache will be of the migraine or tension-type headache type
- Frequent complaints of difficulty concentrating, memory problems, fatigue, hypersensitivity to light and sounds, visual disturbances, dizziness and irritability

Investigation: In the acute phase, a decision is made on the need for a CT or MRI scan of the cerebrum, later there is typically no need for further paraclinical examinations. Treatment: Treat headache according to clinical phenotype [60].

#### Medication overuse headache

See Section “Medication Overuse Headache”.

#### Idiopathic intracranial hypertension (IIH)

Typically seen in younger women of childbearing age with a peak incidence in women at the age of 25 years (15.2 per 100,000) [61].

- By far most common in obese people
- Papilledema is the most prominent feature
- The headache may worsen in the supine position and be worst in the morning
- In addition to headaches, there may be neck pain / back pain, visual field defects, transient visual obscurations, abduction paresis and pulsating tinnitus
- Suspected cases require acute hospitalization (important differential diagnosis: sinus vein thrombosis) and neuroradiological examination, possibly measurement of the cerebrospinal pressure, which will be elevated more than 25 cm H2O
- Untreated intracranial hypertension can lead to permanent visual impairment or blindness

Investigation and treatment: see national neurological treatment guide.

Link: http://neuro.dk/wordpress/nnbv/iih-idiopatisk-intrakraniel-hypertension/

#### Low-pressure headaches

- Typically seen after lumbar puncture but can be seen spontaneously.
- Typical deterioration in an upright position and rapid improvement in a supine position.

Accompanying symptoms in the form of nausea, and possibly paresthesias, tinnitus and dizziness Examination: MRI scan of the cerebrum and columna totalis with contrast.

Treatment: Blood patch (epidural injection of autologous blood) [62].

#### Subarachnoid haemorrhage (SAH)

Acute onset headache (thunderstorm headache):

- May be initiated by generalized seizures
- May be followed by changes in the level of consciousness

Investigation and treatment: see national neurological treatment guide.

Link: https://neuro.dk/wordpress/nnbv/vaskulaere-malformationer-og-sah/

#### Giant cell arteritis (arteritis temporalis)

Typically occurs after the age of 50 with an incidence of 15–44 per 100,000 aged ≥50 years in Northern Europe [63] . Typical symptoms are:

- Headache and general symptoms (e.g. fatigue, fever, night sweats, and weight loss)
- Tenderness on palpation of the temporal artery
- Chewing claudication (up to 40% have this)
- Amaurosis fugax (approximately 10%), which may lead to blindness if left untreated.
- Elevated CRP or erythrocyte sedimentation rate (in rare cases, however, these may be normal)
- Arteria temporalis biopsy positive in 50% of cases. Treatment should be started on clinical suspicion before a possible biopsy response. Contact on-duty doctor in internal medicine or rheumatology in case of suspicion.

#### Primary glaucoma

Headache can be a symptom of narrow-angle glaucoma, which is characterized by:

- Rarely occurring before the age of 50.
- Risk factors: familial disposition, woman and myopia.
- The condition may manifest as acute ocular hypertension.
- Painful red eye.
- Medium dilated pupil without light reaction.
- Accompanied by nausea and vomiting.
- Complains of blurred vision and coloured rings around light objects.

Investigation and treatment: Contact the ophthalmologist on duty urgently in case of suspicion.

#### Cerebral venous thrombosis

(also called sinus vein thrombosis and sinus thrombosis)

Has an overall incidence of 1.75 per 100,000/y with no significant sex differences [64]. Subacute onset, gradually increasing headache is the onset symptom in 70% of cases with fluctuating neurological symptoms:

- Supranuclear palsy (60%)
- Papilledema (30–60%)
- Meningism (25–30%)
- Decreased level of consciousness (60%)
- Epileptic seizures, possibly with Todd’s palsy (40–50%)

Investigation and treatment: see national neurological treatment guide.

Link: https://neuro.dk/wordpress/nnbv/sinustrombose/

#### Arterial dissection

Can be headache that occurs after twisting or trauma to the neck accompanied by neck pain, Horner’s syndrome or atypical aura.

Investigation and treatment: see national neurological treatment guide.

Link: http://neuro.dk/wordpress/nnbv/sjaeldne-arsager-til-apopleksi/

#### Brain tumour

Headache is a frequent symptom of tumour cerebri (60%) but is rarely the only symptom (2%). The overall annual incidence rate of all brain tumours is 7 per 100,000 population [65]. Symptoms can be:

- New onset of headache (tension-type character),
- Worsening of pre-existing headache,
- Headache worst in the morning and accompanied by nausea,
- Possibly with epileptic seizures,
- Cognitive change or personality change,
- Speech disorders and / or hemiparesis.

Investigation and treatment: see national neurological treatment guide.

Link: http://neuro.dk/wordpress/nnbv/primaer-hjernetumor-lavgradsgliom/

#### Neuroinfection

Typical headache accompanied by fever and neck stiffness

- May present with cognitive impairment, photophobia or petechiae
- May present with seizures

Investigation and treatment: see national neurological treatment guide.

Link: https://neuro.dk/wordpress/nnbv/meningitis/

#### Sinusitis

A very common condition [66] with typical headache after upper respiratory tract infection or in pollen season.

Symptoms:

- Pain localization above, behind and under the eyes, aggravation by forward bending.
- Tightness in the nose
- Can present with fever and malaise
- Some patients may experience blurred vision

Investigation: Thorough neurological examination, incl. Ophthalmoscopy and otoscopy. Treatment: Decongestant nasal spray or drops, salt-water nasal rinse or temporary over-the-counter painkillers. In rare cases, antibiotic treatment may be necessary.

#### Other reasons

Headache can also be seen in reversible cerebrovascular vasoconstrictor syndrome (RCVS) (recurrent cases of thunderclap headache), cerebral infarction (accompanied by neurological focal signs, typically from the posterior area of the brain), cerebral, subdural and epidural hematoma, and vascular malformations (atypical malformations)) including e.g. non-ruptured aneurysm, arteriovenous malformation and dural arteriovenous fistula.

## Trigeminal neuralgia

### Diagnosis

Trigeminal neuralgia (TN) is usually located unilaterally and is defined by short-lasting and very painful attacks of stabbing pain in the territory of the trigeminal nerve (Table 19). There can be numerous pain attacks per day. Approximately half of all patients also have concomitant persistent pain in the same area as the stabbing pain. The pain is usually located in the cutaneous and/or mucosal area innervated by the second and third branch of the trigeminal nerve. Pain attacks are elicited by light sensory stimuli such as chewing, touching the face, talking, tooth brushing, shaving and cold wind. In addition, there can be spontaneous pain.

**Table 19:** Clinical characteristics in trigeminal neuralgia

• Trigeminal neuralgia (TN) is a unilateral disorder of short-lasting stabbing pain paroxysms.	
• The painful area typically involves the 2nd and/or 3rd trigeminal branch.	
• The mean age of onset is 52 years, but the range of onset is wide (8–90 years).	
• Pain attacks are evoked by light sensory stimuli such as chewing, touching the face, talking, tooth brushing, shaving and cold wind. There can also be spontaneous pain.	
• Trigger zones are often located around the nasal wing and the lateral part of the upper and lower lip.	
• Natural history of TN is unpredictable. There may be severe exacerbations of pain and there may be periods of complete pain remission lasting for weeks and months – in some cases even years.	
• Symptomatic TN is caused by a brainstem plaque from multiple sclerosis or by a space-occupying lesion in the cerebellopontine angle cistern. At clinical presentation, it can be indistinguishable from primary TN.	

At clinical examination, there is typically trigger zones located around the nasal wing and the lateral part of the upper and lower lip [67]. At disease onset, the pain is often misdiagnosed as originating from the teeth and sinuses. The natural history of TN pain is usually very unpredictable. Severe pain exacerbations may warrant admittance to a neurological ward to treat pain, dehydration and anorexia. At the other end of the spectrum, there may be periods of complete pain remission lasting for weeks and months – in some cases even years. Sporadic ipsilateral autonomic symptoms may occur in relation to severe pain attacks.

The diagnostic criteria according to the 3rd edition of the International Classification of Headache Disorders (ICHD-3) are shown in Table 20 [2]. Persisting idiopathic facial pain (previously termed” atypical facial pain”) is dominated by constant and more diffusively located facial pain. It is less common for patients to have trigger factors. There may be TN-like trigger factors such as sensory stimuli to the affected side of the face, however, just as often, “trigger factors” may be physical or psychological stress [68].

**Table 20:** Diagnostic criteria in trigeminal neuralgia [2]

A. Recurrent paroxysms of unilateral facial pain in the distribution(s) of one or more divisions of the trigeminal nerve, with no radiation beyond, and fulfilling criteria B, C and D	
B. Pain has all of the following characteristics:	
1. lasting from a fraction of a second to 2 min	
2. servere intensity	
3. electric shock-like, shooting, stabbing or sharp in quality	
C. Precipitated by innocuous stimuli within the affected trigeminal distribution	
D. Not better accounted for by another ICHD-3 diagnosis	

### Background

TN onset is approximately 52 years and TN affects more women than men. TN is diagnosed according to the international diagnostic criteria (Table 20) and is then subsequently sub-grouped into primary (classical and idiopathic), and symptomatic TN based on MRI-findings. Classical TN is diagnosed when there is a neurovascular contact between the trigeminal nerve and a blood vessel in the cerebellopontine angle cistern causing morphological changes of the trigeminal nerve such as dislocation, distortion or atrophy. In idiopathic TN, there is either no neurovascular contact or there is a neurovascular contact without morphological changes of the trigeminal nerve. Symptomatic TN can be caused by a brainstem plaque in a patient with multiple sclerosis or by a space-occupying lesion in the cerebellopontine angle cistern.

A neurovascular contact without morphological changes of the trigeminal nerve is a frequent neuroanatomical finding in healthy individuals [69]. Therefore, a neurovascular contact is not necessarily, what causes pain in the individual patient. No matter the disease ethology, it is presumed that the pathophysiological process leading to TN pain, is defined by demyelination of the trigeminal nerve paving the way for ephaptic impulses and cross-excitation between neighbouring sensory axons [70].

### Clinical assessment

A thorough history and a detailed neurological examination are essential to set a correct diagnosis. It is particularly important to rule out whether the pain was caused by a trauma, e.g. invasive odontogenic treatment. In that case, the diagnosis is painful posttraumatic trigeminal neuropathy. Sensory abnormalities and bilateral pain may indicate symptomatic TN, although these clinical characteristics are also seen in classical and idiopathic TN [71]. In approximately 15% of TN patients, there is an underlying symptomatic cause of pain (not including a neurovascular contact). It is not possible to accurately identify all patients with symptomatic TN based on pain characteristics, clinical examination or treatment response. Therefore, an MRI of the brain and brainstem is mandatory early on during work-up. MRI should focus on exclusion of a symptomatic cause of pain and on visualization and characterization of a potential neurovascular contact. Scanning sequences should include multiple thin slices and angiography focused on the cerebellopontine angle cistern. Three point zero T MRI identifies more neurovascular contacts compared to 1.5 T MRI, but 1.5 T MRI is sufficient to rule out a symptomatic cause of pain [71].

### Non-pharmacological treatment

Some patients report that acupuncture has a good efficacy; however, there is no scientific evidence to support acupuncture or other non-pharmacological treatments for TN.

### Pharmacological treatment

#### Acute treatment

Over-the-counter analgesics and opioids usually have a poor efficacy in TN. At severe pain exacerbations, intravenous infusion of either phenytoin or lidocaine may be undertaken during hospitalization. Both treatments are highly specialized and continuous monitoring is mandatory.

#### Preventive treatment

Preventive medical treatment has a stabilizing effect on the trigeminal nerve [72]. Carbamazepine or oxcarbazepine are first choice medical treatments. According to clinical experience, it is difficult to predict whether the individual patient will benefit the most and have the fewest medical side effects on one or the other of these two drugs. Therefore, if one drug is inefficacious or poorly tolerated, the other drug may be tried out. To switch between the two, it is possible to switch directly to the equipotent dose of the other drug (200 mg carbamazepine = 300 mg oxcarbazepine). Oxcarbazepine is usually better tolerated compared to carbamazepine, but oxcarbazepine has a greater tendency to induce hyponatremia. Cross-allergic reactions are seen in 25% of patients.

Gabapentin, pregabalin and lamotrigine may be used as add on treatments or as monotherapy if the first choice treatments are not tolerated. A suggested treatment strategy is to titrate carbamazepine or oxcarbazepine to the highest tolerated dose and then add on gabapentin, pregabaline or lamotrigine and titrate to a tolerated and sufficient dose.

Botulinum toxin A may be efficacious and is specialist treatment.

As TN usually has an unpredictable pattern of pain frequency and intensity, dose(s) of medical treatment should be titrated and tapered according to pain level. At complete pain freedom lasting more than 1 month, it is advised to taper off medication by reducing e.g. carbamazepine by 100 mg or gabapentin by 300 mg every 7th–14th day (or comparable doses of other TN drugs) [73]. Symptomatic TN is treated medically according to the same guidelines as classical and idiopathic TN [71].

##### Drugs for trigeminal neuralgia

1. Carbamazepine, optionally slow-release preparation. First choice treatment [73].
  Efficacy: only medical treatment with documented efficacy in several controlled studies. Approximately 60–70% achieve a 50% reduction in level of pain. The efficacy is often limited by side effects.
  Initial dose is 100–200 mg BID. Titrate 100 mg every 3rd day until pain freedom or unacceptable side effects
  Typical maintenance dose is 100–600 mg BID
  Daily doses of 1800 mg or more may be necessary

2. Oxcarbazepine. First choice treatment [73].
  Efficacy: comparable to the efficacy of carbamazepine [71].
  Initial dose is 150–300 mg BID. Titrate 150 mg every 3rd day until pain freedom or unacceptable side effects
  Typical maintenance dose is 150–900 mg BID
  Daily doses of 2700 mg or more may be necessary

3. Gabapentin. Add on to first choice treatments or monotherapy [73].
  Efficacy: low level of scientific evidence on efficacy but there is consensus among specialists that the drug is effective in TN.
  Initial dose is 300 mg daily titrating with 300 mg every 3rd day until efficacy or unacceptable side effects. Maximum daily dose is 3600 mg divided in three doses.
  Typical maintenance dose is between 300 mg BID up to 1200 mg TID.

4. Pregabaline. Add on to first choice treatments or monotherapy [73].
  Efficacy: low level of scientific evidence on efficacy but there is consensus among specialists that the drug is effective in TN.
  Initial dose is 75 mg BID titrating with 150 mg every 7th day until efficacy or unacceptable side effects. Maximum daily dose is 600 mg divided in two doses.
  Typical maintenance dose is 75 mg - 300 mg BID.

5. Lamotrigine. Add on to first choice treatments or monotherapy [73].
  Efficacy: low level of scientific evidence on efficacy but there is consensus among specialists that the drug is effective in TN.
  Initial dose is 25 mg daily for 2 weeks, then 50 mg daily for 2 weeks and hereafter titrating with 50 mg per week until 50 mg BID. Observe efficacy. The drug can be further titrated with 50 mg per week until efficacy or unacceptable side effects.
  Typical maintenance dose is 50 mg - 200 mg BID.

For an in-depth description of TN medical treatment, we refer to a recent review by Bendtsen et al. [73].

### Neurosurgical treatment

Approximately 30% of all TN patients do not have sufficient effect from medical treatment or have unacceptable medical side effects. In that case, neurosurgical treatment should be considered.

#### Microvascular decompression

Microvascular decompression is the most effective surgical treatment in TN. The procedure is performed via a retromastoid craniotomy. Blood vessels in contact with the trigeminal nerve are transposed away from the nerve. Initially, 90% of patients has a good efficacy. After 1 and 5 years efficacy drops to 80% and 73%, respectively [71]. The perioperative mortality ranges from 0.2–0.5% and 4% are affected by serious surgical complications such as hematoma, ischemic infarction, and leakage of cerebrospinal fluid. The most frequent serious complication is ipsilateral hearing loss (10%). Dysesthetic pain and hypoesthesia can also occur. Several studies indicate that the chance of a good surgical outcome is dependent on the presence of preoperative morphological changes of the affected trigeminal nerve [71]. One study found that men have a higher chance of a good surgical outcome compared to women [74].

#### Percutaneous procedures

Percutaneous procedures are recommended in patients who do not have a neurovascular contact, where surgery or anaesthesia is contraindicated or if the patient prefer percutaneous procedures over microvascular decompression. Parts of the trigeminal ganglion are destructed chemically (glycerol injection), thermically (thermocoagulation) or mechanically (balloon compression). Approximately 68–85% of patients have a good efficacy after 1 year and after 5 years, 50% remains to have a good efficacy [73]. Sensory changes such as hypoesthesia, paresthesia and dysesthesia are seen in more than 50% of patients postoperatively. Sensory changes will often improve with time. About 4% of patients develop painful dysesthesia (anaesthesia dolorosa) [71]. In conclusion, percutaneous procedures are less invasive compared to microvascular decompression, but the rate of success is lower and the recurrence rate is higher.

### Summary

Trigeminal neuralgia (TN) is a unilateral disorder characterized by ultra-short attacks of stabbing pain located in one or more of the trigeminal branches. Attacks are elicited by sensory stimuli such as chewing, talking, and brushing teeth. TN may be caused by a neurovascular contact with vascular compression of the trigeminal nerve in the prepontine segment of the nerve. MRI is a mandatory part of early work-up. The treatment is preventive medical treatment med antiepileptic drugs. If the medial efficacy is insufficient or the medical side effects are unacceptable, neurosurgical treatment of either microvascular decompression or percutaneous procedures must be considered. A close co-operation between neurologists, neuroradiologists and neurosurgeons is essential to optimal treatment of this highly painful condition [71, 73].

## Hormones and migraines

This section describes the specific conditions that apply to migraines in relation to menstruation, hormone therapy, pregnancy and breastfeeding.

### Diagnosis

Menstrual migraine is defined as migraine attacks that occur on the first day of menstruation ±2 days for at least 2 out of 3 menstrual cycles. By menstruation is meant endometrial bleeding which originates either from the normal menstrual cycle or from discontinuation of added female sex hormone, e.g. from oestrogen-containing contraceptive pills and cyclic hormone therapy. The vast majority of women also have migraine attacks at times other than in relation to the menstrual cycle. Migraine is classified in these cases as menstrual related migraine. If migraine attacks occur exclusively during menstruation, this is defined as pure menstrual migraine [2].

### Background

The incidence of migraine is related to the menstrual cycle, this is especially true for migraine without aura. Thus, migraines are equally prevalent in girls and boys before puberty, whereas approximately three times as many women as men suffer from migraines after puberty. Migraine can be triggered by a sudden drop in oestrogen levels, but only if prior to this there has been a high oestrogen level for several days. This explains why migraines occur with increased frequency around menstruation and with decreased frequency during pregnancy, while there is no definite relationship to ovulation. Around the menopause, a number of women experience worsening of the migraine. After menopause, both the incidence and prevalence of migraine decrease [75].

### Specific risk factors

There is a slightly increased risk of myocardial infarction and both ischemic and haemorrhagic stroke in migraine with aura [76, 77]. Several case-control, cohort studies and pooled data analyses indicate that the risk of cerebral infarction is increased by 1.5–2 times in people with migraine with aura, while there is no increased risk in people with migraine without aura [78]. The risk is present in women under 45 years. However, if older women continue to have migraines and smoke at the same time, it seems to increase the risk of stroke [79]. However, the absolute risk of cerebral infarction is small, approximately 0.006% in women with migraine with aura in relation to the background population (0.0025%). However, the risk more than doubles if these women take oestrogen-containing birth control pills (0.015%) and the risk increases in women who smoke [78]. Women with migraines with aura should be encouraged not to smoke. Women with migraines with aura should be informed that they have a slightly increased risk of an ischemic stroke in the brain, but that the risk is very small if there are no other risk factors and if they refrain from smoking and taking oestrogen-containing birth control pills. Thus, for the vast majority of patients, migraine is a benign, albeit debilitating, disease.

### Choice of contraception

#### Migraine with aura

If contraception is needed in women with migraine with aura, birth control pills with the lowest possible oestrogen content are preferred, and the patient must be informed of the increased risk of ischemic stroke. High-risk preparations: Contraceptive pills with oestrogen content ≥35 μg, medium-risk preparations: Contraceptive pills with oestrogen content < 35 μg, oestrogen patches and vaginal ring are NOT recommended for women with migraines with aura. Instead, hormonal contraception is recommended which does NOT increase the risk of ischemic stroke, i.e. preparations containing exclusively progestogens: mini-pills, subdermal implant (contraceptive stick), depot injection and IUD.

#### Migraine without aura

Sometimes patients experience aggravation of migraine without aura by using oestrogen-containing birth control pills, other patients experience no changes in their headaches and finally some patients can experience an improvement of the headache.

In case of need for contraception, where a worsening of migraine without aura is experienced at the same time, the following can be tried:

- Use of oestrogen-containing contraceptive pills, where there is no contraceptive pill break through several cycles, e.g. by taking birth control pills continuously for 9 weeks (instead of the usual 3 weeks) followed by a 7 day pill-free period. If breakthrough bleeding occurs earlier, there is a pause at the time of breakthrough bleeding.
- Use of mini-pills (containing desogestrel 75 μg / day only).

There are no special precautions in women with migraine without aura or non-migraine headache. For women who have vascular risk factors: smoking, hypertension, obesity, previous cardiovascular event or previous deep vein thrombosis, birth control pills with oestrogen content ≤35 μg, oestrogen patches and vaginal ring are used.

Currently, only observational studies on desogestrel are available. There are no scientific studies describing the effect on migraine or the risk of blood clots when using norethisterone, levonorgestrel, non-oral modalities such as subdermal implant (IUD), depot injection or IUD. Two observational studies compare the above regimens and suggest that mini-pills may be more effective in preventing migraines [80].

### Treatment of menstrual migraines

The principles of non-pharmacological treatment and attack treatment of menstrual migraine do not differ from the treatment of non-hormone-related migraine. However, menstrual migraines are more often more difficult to treat than non-menstrual migraines. If the menstrual cycle is completely regular, one can try short-term cyclic prevention, which begins 2 days before the first menstrual day and is given for 6 days.

The following triptans have been found to be effective as short-term cyclic prevention, taken twice daily: frovatriptan, sumatriptan, zolmitriptan and naratriptan [75, 81]. However, be aware of the risk of medication overuse headaches. The number of days per month of triptan consumption should not exceed nine.

- NSAIDs, e.g. Naproxen 500 mg × 2 tablets.
- Magnesium tablet 360–400 mg × 1 which is taken daily from day 15 of the cycle (calculated from the first day of menstruation) can also be tried [75, 81].

Short-term dose escalation of common preventive medication may be attempted.

Other strategies have been explored. It is uncertain whether phytoestrogens, which are substances found in plants whose structure resembles human oestrogen, have an effect on menstrual migraines. The same goes for vitamin E supplements. Acupuncture has been studied and has not been shown to have an effect on menstrual migraines [75, 81].

### Pregnancy

Most patients experience an improvement in migraines during pregnancy, which most often sets in in the second trimester, but some women experience worsening or more frequent attack, especially at the end of the first trimester where HCG levels fall. During pregnancy where oestrogen levels are high, migraine with aura can start, and relatively often a severe migraine without aura can occur immediately after birth, provoked by the sharp decrease in oestrogen levels [82]. In the vast majority, migraine recur after birth or cessation of breastfeeding.

Attack treatment during pregnancy

- As far as possible non-pharmacological with calm, bed rest, ice packs and the like.
- If drug treatment is needed, paracetamol is the first choice.
- NSAIDs should be avoided.
- Sumatriptan can be used if necessary. The other triptans are not recommended due to sparse data.
- Metoclopramide can be used. Domperidone is not recommended.
- Ergotamine is contraindicated due to the uterine contracting effect [82].

Preventive treatment

Preventive treatment should be avoided if possible.

- As far as possible non-pharmacological: regular lifestyle, incl. Regular nutritious meals, sleep, physical activity and tranquillity.
- Beta-blockers can be used in the lowest possible dosage, but there is a risk of side effects in the newborn such as bradycardia, hypotension and hypoglycaemia.
- Antiepileptic drugs and antidepressants are not recommended [82].

### Breastfeeding

Attack treatment

- As far as possible non-pharmacological with calm, bed rest, ice packs and the like.
- If the above has been tried and is insufficient, paracetamol can be used.
- NSAIDs can be used; ibuprofen is preferable (due to short half-life, no active metabolites and low concentration in breast milk).
- Sumatriptan and eletriptan can be used if necessary. Breast-feeding is not recommended for 12 h after ingestion of other triptans.
- Caution with metoclopramide as it is absorbed into breast milk.
- Avoid acetylsalicylic acid, benzodiazepines and ergotamine.

Preventive treatment

- Avoid drug prevention as much as possible.
- Beta-blockers, valproate and amitriptyline may be used [82].

## Children and headaches

### Diagnosis

Diagnosis of headache in children follows the ICHD-3 criteria used in adults, with minor modifications (added under notes in Table 21). This section focuses on the characteristics that are specific to children. The classification of headaches in children is given in Table 21.

**Table 21:** Classification of headache in children [2]

1.1 [G43.1b] Migraine without aura	
A. At least five attacks fulfilling criteria B-D.	
B. Headache attacks lasting 2–72 h (untreated or unsuccessfully treated)	
C. Headache at least two of the following four characteristics:	
1. unilateral location	
2. pulsating quality	
3. moderate or severe pain intensity	
4. aggravation by or causing avoidance of routine physical activity (e.g. walking or climbing stairs)	
D. During headache at least one of the following:	
1. nausea and/or vomiting	
2. photophobia and phonophobia	
E. Not better accounted for by another ICDH-3 diagnosis	
1.1 [G43.1b] migraine with aura	
Children and adults share the same diagnostic criteria	
1.6.1.1 [G43.1B] Cyclical vomiting syndrome	
A. At least five attacks of intense nausea and vomiting, fulfilling criteria B and C	
B. Stereotypical in the individual patient and recurring with predictable periodicity	
C. All of the following:	
1. nausea and vomiting occur at least four times per hour	
2. attacks last for ≥1 h, up to 10 days	
3. attacks occur ≥1 week apart	
D. Complete freedom from symptoms between attacks	
E. Not attributed to another disorder	
1.6.1.2 [G43.1B] Abdominal migraine	
A. At least five attacks of abdominal pain, fulfilling criteria B–D	
B. Attacks last 2–72 h when untreated or unsuccessfully treated	
C. Pain has at least two of the following three characteristics:	
1. midline location, periumbilical or poorly localized	
2. dull or “just sore” quality	
3. moderate or severe intensity	
D. At least two of the following four associated symptoms or signs:	
1. anorexia	
2. nausea	
3. vomiting	
4. pallor	
E. Complete freedom from symptoms between attacks	
F. Not attributed to another disorder	
1.6.2. [G43.1B] Benign paroxysmal vertigo	
A. At least five attacks fulfilling criteria B and C	
B. Vertigo occurring without warning, maximal at onset and resolving spontaneously after minutes to hours without loss of consciousness	
C. At least one of the following five associated symptoms or signs:	
1. nystagmus	
2. ataxia	
3. vomiting	
4. pallor	
5. fearfulness	
D. Normal neurological examination and audiometric and vestibular functions between attacks	
E. Not attributed to another disorder	
[G44.2] Tension-type headache	
Children and adults share the same diagnostic criteria	

Comments:

1. Migraine is often usually bilateral in young children; Unilateral headache typically occurs in late adolescence or early adulthood.

2. Pain location is often frontotemporal. Occipital headache in children, whether unilateral or bilateral, is rare and requires diagnostic caution as it may be due to structural lesions.

3. In young children, light and sound hypersensitivity can typically be detected by observing the children’s reaction pattern.

4. Cyclic vomiting syndrome is an exclusion diagnosis. History, clinical and neurological examination must not give rise to suspicion of other diseases. Thorough diagnostic examination is always necessary with regard to exclusion of other disease. Differential diagnoses: intermittent bowel obstruction (malrotation), kidney, liver, pancreatic disease, elevated intracranial pressure, poisoning, metabolic disease and epilepsy.

5. In abdominal migraine patient history, clinical and neurological examination should not raise suspicion of gastrointestinal or kidney disease or these disorders should be ruled out by proper examination.

6. In benign paroxysmal vertigo, especially young children cannot describe vertigo, but may be idnetified by gait difficulties. It is always important to rule out fossa posterior tumors, epilepsy and vestibular disease

### Background

Migraine in children differs from migraine in adults especially by:

- Shorter attack duration,
- More frequent bilateral localization
- Pronounced gastrointestinal symptoms.

The special periodic syndromes that occur in children (Cyclic Vomiting Syndrome, Abdominal Migraine and Benign Paroxysmal Vertigo) may be precursors to migraines in adulthood, but they are relatively rare conditions. Other primary forms of headache, such as tension-type headache and cluster headache, can also start in early childhood, although onset is more frequently seen around puberty, but does not differ from the symptom picture in adults. Medication overuse headache also exists in children and adolescents, but does not differ from medication overuse headache in adults.

### Clinical assessment

The headache diary is an important diagnostic tool (see Fig. 1, can be downloaded at dhos.dk).

In general, it applies to:

- Older children and young people can use the headache diary without any problems.
- Most 7–11-year-olds can self-report pain frequency and intensity but may depend on an adult to record their other symptoms.
- In young children, parents can observe and report their children’s symptoms [83] and the intensity of the pain can be determined by children, using a visual analog scale.

All children with headaches should have a complete objective and neurological examination. The objective examination should also include measurement of blood pressure and heart rate and in some cases an eye examination including examination of eye background. These tests as well as any other diagnostic tests are done primarily to rule out other causes of the child’s headache. It should be noted that brain tumours in children, as opposed to in adults, most often is localized in the infratentorial region of the brain in or adjacent to the cerebellum and can therefore often cause balance problems.

### Treatment

In general, children are treated according to the same treatment principles as adults but taking into account the limited results that exist from randomized placebo-controlled studies in children and adolescents.

#### Non-pharmacological treatment

Non-pharmacological treatment should always be attempted before initiating drug treatment, but the evidence base is extremely limited (Table 22).

**Table 22:** Non-pharmacological treatment of headache in children

Non-pharmacological treatment of headache in children	
• Objective examination and reassurance.	
• Exclude other underlying disorder e.g. stress, psychogenic factors (problems at home, school or / and among peers), depression, depression, anxiety, refraction anomalies, strabismus, over-strained eyes (computer work / games), oromandibular dysfunction, sinusitis, posture anomaly, passive / active smoking and inappropriate lifestyle.	
• Exclude medication-overuse headache.	
• Inform about disease mechanisms so that both child and parents understand it.	
• Minimize or eliminate triggers, e.g. stress or poor posture during schoolwork.	

Non-pharmacological treatment includes:

- Identification and elimination / reduction of provocative headache trigger factors.
- Talk to the child about fluid intake, regular meals / diets and sleep patterns.
- Talk to the child about excessive TV-screen time and lack of physical activity.
- Talk to the child about any stressors / pain management. In case of stress and / or pain, biofeedback / relaxation and cognitive therapy for pain coping is used.
- Identify possible comorbidity (focus points are attention problems, social difficulties, professional difficulties, family challenges / illness) - as unidentified comorbidity can cause stress. In case of symptoms of stress, relevant investigation / help is initiated.
- Identify any dental or back problems - initiate appropriate help.
- Identify any vision problems - initiate relevant help.
- Identify medication overuse headache.
- Thorough information of children / youth and their parents regarding conclusion and plan for treatment. In addition, it is important to minimize their possible concern about serious illness - when there is no evidence of this.

#### Pharmacological treatment

Pharmacological treatment of headache diseases in children is mostly based on treatment principles based on studies in adults (Table 23). That is generally inadequate evidence in relation to both acute and preventive treatment of headache diseases in children and adolescents and there is a great need for further randomized placebo-controlled trials (RCTs).

**Table 23:** Pharmacological treatment of headache in children

Pharmacological treatment of headache in children	
• Treatment of acute tension headache attacks (paracetamol and / or NSAIDs).	
• Treatment of acute migraine attacks (paracetamol and / or ibuprofen possibly combined with domperidone (at age > 12 years and weight > 35 kg)), alternatively (sumatriptan nasal spray (children> 12 years) or tablet zolmitriptan (children> 12 years) possibly in combination with ibuprofen).	
• Generally avoid overuse of painkillers.	
• Preventive treatment is considered for very frequent or severely disabling headaches, where there has been insufficient effect of the non-pharmacological treatment and where the acute seizure treatment is inadequate and medication overuse headache is excluded.	
• Beta-blockers and flunarizine have some proven prophylactic effect, but in general, there is very little scientific evidence that prophylactic medical treatment has an effect in children with migraines and tension headaches.	
• If prophylactic treatment is needed, the general rules for adults are followed.	

##### Acute migraine attack treatment

A few less randomized placebo-controlled studies have documented the efficacy of ibuprofen and the lack of effect of paracetamol [84, 85]. Monotherapy with other NSAIDs (naproxen, ketoprofen, diclofenac and indomethacin) has not been tested in children / adolescents in RCTs.

RCTs have shown significant efficacy, tolerability and safety when treated with different triptans. Triptans appear to be more effective than placebo, but the results are variable and inconsistent. Almotriptan, rizatriptan and (sumatriptan / naproxen in combination) are effective as oral formulations; while sumatriptan and zolmitriptan are only proven effective (in monotherapy) as a nasal spray [86]. Addition of NSAIDs enhances the effect of triptan [86]. In Denmark, sumatriptan nasal spray and zolmitriptan tablet are approved for children / adolescents> 12 years.

Paracetamol and ibuprofen have fewer side effects than triptans [86]. Triptan-related side effects in children / adolescents are comparable to side effects observed in adults. Unpleasant taste is the most common side effect with nasal spray [86]. In case of nausea, the above treatment can be supplemented with domperidone (only for weight > 35 kg and age > 12 years). Domperidone is used as an antiemetic rather than metoclopramide due to the lower risk of movement disorders.

##### Acute attack treatment of tension-type headache

The effects of paracetamol, NSAIDs and combinations of codeine/aspirin have been documented, but codeine/aspirin combinations are not recommended for use in children below 15 years of age due to the risk of Reye’s syndrome.

### Preventive treatment

There is only an indication for pharmacological preventive treatment of headaches in children if the non-pharmacological treatment and acute treatment are ineffective and / or the headache attacks are frequent (more than 3–4 days per month), long and / or so severe that the seizures significantly influence quality of life and / or functional level.

#### Prevention of migraine

Treatment with beta-blockers (propranolol or metoprolol) and flunarizine has a comparable preventive effect in children and adolescents [87, 88]. The effect of beta-blockers has been documented in a single RCT and flunarizine in a single randomized study [87, 88]. There are need more RCTs to document an effect of beta-blockers and flunarizine. Valproate has a preventive effect in controlled studies, but the effect is lower than for beta-blockers and is associated with several side effects. Amitriptyline and topiramate have no documented effect in children [89]. With the recent development of calcitonin gene-related peptide (CGRP) antagonist treatment, which appears to have an effect and good safety profile in adults, it can be hoped that these new types of preventive treatment also play a role in the preventive treatment of migraines in children. See Section “Migraine” for more information on CGRP antibodies.

#### Prevention of chronic tension headache

Prevention with amitriptyline may have an effect on chronic tension-type headaches in children, but there are no placebo-controlled studies.

## Data Availability

All included references in the present review article are available on the Internet.

## References

[1] GBD (2019). Diseases and injuries collaborators (2020) global burden of 369 diseases and injuries in 204 countries and territories, 1990-2019: a systematic analysis for the global burden of disease study 2019. Lancet.

[2] Headache Classification Committee of the International Headache Society (IHS) (2018) The International Classification of Headache Disorders, 3rd edition. Cephalalgia 38:1–211. 10.1177/0333102417738202 10.1177/0333102417738202 29368949

[3] Russell MB, Ulrich V, Gervil M, Olesen J (2002). Migraine without aura and migraine with aura are distinct disorders. A population-based twin survey. Headache.

[4] Rasmussen BK, Jensen R, Schroll M, Olesen J (1991). Epidemiology of headache in a general population--a prevalence study. J Clin Epidemiol.

[5] Ashina M (2020). Migraine. N Engl J Med.

[6] Nestoriuc Y, Martin A, Rief W, Andrasik F (2008). Biofeedback treatment for headache disorders: a comprehensive efficacy review. Appl Psychophysiol Biofeedback.

[7] Linde K, Allais G, Brinkhaus B et al (2016) Acupuncture for the prevention of episodic migraine. Cochrane Database Syst Rev:CD001218. 10.1002/14651858.CD001218.pub3. 10.1002/14651858.CD001218.pub3 PMC4977344 27351677

[8] Luedtke K, Allers A, Schulte LH, May A (2016). Efficacy of interventions used by physiotherapists for patients with headache and migraine-systematic review and meta-analysis. Cephalalgia.

[9] Tfelt-Hansen P, Henry P, Mulder LJ, Scheldewaert RG, Schoenen J, Chazot G (1995). The effectiveness of combined oral lysine acetylsalicylate and metoclopramide compared with oral sumatriptan for migraine. Lancet.

[10] Evers S, Afra J, Frese A, Goadsby PJ, Linde M, May A, Sándor PS, European Federation of Neurological Societies (2009). EFNS guideline on the drug treatment of migraine--revised report of an EFNS task force. Eur J Neurol.

[11] Tfelt-Hansen PC (2017). Delayed absorption of many (paracetamol, aspirin, other NSAIDs and zolmitriptan) but not all (sumatriptan, rizatriptan) drugs during migraine attacks and most likely normal gastric emptying outside attacks. A review. Cephalalgia.

[12] Scholpp J, Schellenberg R, Moeckesch B, Banik N (2004). Early treatment of a migraine attack while pain is still mild increases the efficacy of sumatriptan. Cephalalgia.

[13] Olesen J, Diener HC, Schoenen J, Hettiarachchi J (2004). No effect of eletriptan administration during the aura phase of migraine. Eur J Neurol.

[14] Schytz HW, Bendtsen L (2014) Sumatriptan plus naproxen for acute migraine attacks in adults. Ugeskr Laeger 176(32):V03140155. 25292477

[15] Schulman EA, Dermott KF (2003). Sumatriptan plus metoclopramide in triptan-nonresponsive migraineurs. Headache.

[16] Mathew PG, Krel R, Buddhdev B, Ansari H, Joshi SG, Spinner WD, Klein BC (2016). A retrospective analysis of triptan and dhe use for basilar and hemiplegic migraine. Headache.

[17] Artto V, Nissila M, Wessman M (2007). Treatment of hemiplegic migraine with triptans. Eur J Neurol.

[18] Amin FM, Asghar MS, Hougaard A, Hansen AE, Larsen VA, de Koning PJH, Larsson HBW, Olesen J, Ashina M (2013). Magnetic resonance angiography of intracranial and extracranial arteries in patients with spontaneous migraine without aura: a cross-sectional study. Lancet Neurol.

[19] Asghar MS, Hansen AE, Kapijimpanga T, van der Geest RJ, van der Koning P, Larsson HBW, Olesen J, Ashina M (2010). Dilation by CGRP of middle meningeal artery and reversal by sumatriptan in normal volunteers. Neurology.

[20] Kangasniemi P, Hedman C (1984). Metoprolol and propranolol in the prophylactic treatment of classical and common migraine. A double-blind study. Cephalalgia.

[21] Stovner LJ, Linde M, Gravdahl GB, Tronvik E, Aamodt AH, Sand T, Hagen K (2014). A comparative study of candesartan versus propranolol for migraine prophylaxis: a randomised, triple-blind, placebo-controlled, double cross-over study. Cephalalgia.

[22] Bendtsen L, Sacco S, Ashina M, Mitsikostas D, Ahmed F, Pozo-Rosich P, Martelletti P (2018). Guideline on the use of onabotulinumtoxinA in chronic migraine: a consensus statement from the European headache federation. J Headache Pain.

[23] Sacco S, Bendtsen L, Ashina M, Reuter U, Terwindt G, Mitsikostas DD, Martelletti P (2019). European headache federation guideline on the use of monoclonal antibodies acting on the calcitonin gene related peptide or its receptor for migraine prevention. J Headache Pain.

[24] Welch KM, Ellis DJ, Keenan PA (1985). Successful migraine prophylaxis with naproxen sodium. Neurology.

[25] (2019) Global, regional, and national burden of neurological disorders, 1990–2016: a systematic analysis for the Global Burden of Disease Study 2016. Lancet Neurol 18:459–480. 10.1016/S1474-4422(18)30499-X 10.1016/S1474-4422(18)30499-X PMC6459001 30879893

[26] Bendtsen L, Ashina S, Moore A, Steiner TJ (2016). Muscles and their role in episodic tension-type headache: implications for treatment. Eur J Pain.

[27] Bendtsen L, Evers S, Linde M, Mitsikostas DD, Sandrini G, Schoenen J, EFNS (2010). EFNS guideline on the treatment of tension-type headache - report of an EFNS task force. Eur J Neurol.

[28] Linde K, Allais G, Brinkhaus B (2016). Acupuncture for the prevention of tension-type headache. Cochrane Database Syst Rev.

[29] Bendtsen L, Jensen R (2011). Treating tension-type headache -- an expert opinion. Expert Opin Pharmacother.

[30] Jackson JL, Shimeall W, Sessums L, DeZee KJ, Becher D, Diemer M, Berbano E, O'Malley PG (2010). Tricyclic antidepressants and headaches: systematic review and meta-analysis. BMJ.

[31] Lund N, Barloese M, Petersen A, Haddock B, Jensen R (2017). Chronobiology differs between men and women with cluster headache, clinical phenotype does not. Neurology.

[32] Snoer A, Lund N, Beske R, Hagedorn A, Jensen RH, Barloese M (2018). Cluster headache beyond the pain phase: a prospective study of 500 attacks. Neurology.

[33] Barloese M, Haddock B, Lund NT, Petersen A, Jensen R (2018). Chronorisk in cluster headache: a tool for individualised therapy?. Cephalalgia.

[34] Fischera M, Marziniak M, Gralow I, Evers S (2008). The incidence and prevalence of cluster headache: a meta-analysis of population-based studies. Cephalalgia.

[35] Barloese M, Lund N, Petersen A, Rasmussen M, Jennum P, Jensen R (2015). Sleep and chronobiology in cluster headache. Cephalalgia.

[36] May A, Bahra A, Buchel C (1998). Hypothalamic activation in cluster headache attacks. Lancet.

[37] Arkink EB, Schmitz N, Schoonman GG, van Vliet JA, Haan J, van Buchem MA, Ferrari MD, Kruit MC (2017). The anterior hypothalamus in cluster headache. Cephalalgia.

[38] Goadsby PJ (2002). Pathophysiology of cluster headache: a trigeminal autonomic cephalgia. Lancet Neurol.

[39] May A, Leone M, Afra J, Linde M, Sándor PS, Evers S, Goadsby PJ, EFNS Task Force (2006). EFNS guidelines on the treatment of cluster headache and other trigeminal-autonomic cephalalgias. Eur J Neurol.

[40] Petersen AS, Barloese MC, Jensen RH (2014). Oxygen treatment of cluster headache: a review. Cephalalgia.

[41] Petersen AS, Barloese MC, Lund NL, Jensen RH (2017). Oxygen therapy for cluster headache. A mask comparison trial. A single-blinded, placebo-controlled, crossover study. Cephalalgia.

[42] Hoffmann J, May A (2018). Diagnosis, pathophysiology, and management of cluster headache. Lancet Neurol.

[43] Jurgens TP, Barloese M, May A (2017). Long-term effectiveness of sphenopalatine ganglion stimulation for cluster headache. Cephalalgia.

[44] Chan C, Goadsby PJ (2020). CGRP pathway monoclonal antibodies for cluster headache. Expert Opin Biol Ther.

[45] Tassorelli C, Jensen R, Allena M, de Icco R, Sances G, Katsarava Z, Lainez M, Leston JA, Fadic R, Spadafora S, Pagani M, Nappi G, the COMOESTAS Consortium (2014). A consensus protocol for the management of medication-overuse headache: evaluation in a multicentric, multinational study. Cephalalgia.

[46] Munksgaard SB, Bendtsen L, Jensen RH (2012). Treatment-resistant medication overuse headache can be cured. Headache.

[47] Carlsen LN, Munksgaard SB, Jensen RH, Bendtsen L (2018). Complete detoxification is the most effective treatment of medication-overuse headache: a randomized controlled open-label trial. Cephalalgia.

[48] Zwart JA, Dyb G, Hagen K (2004). Analgesic overuse among subjects with headache, neck, and low-back pain. Neurology.

[49] Westergaard ML, Glumer C, Hansen EH (2014). Prevalence of chronic headache with and without medication overuse: associations with socioeconomic position and physical and mental health status. Pain.

[50] Grande RB, Aaseth K, Benth JS, Lundqvist C, Russell MB (2011). Reduction in medication-overuse headache after short information. The Akershus study of chronic headache. Eur J Neurol.

[51] Bigal ME, Serrano D, Buse D, Scher A, Stewart WF, Lipton RB (2008). Acute migraine medications and evolution from episodic to chronic migraine: a longitudinal population-based study. Headache.

[52] Munksgaard SB, Bendtsen L, Jensen RH (2013). Modulation of central sensitisation by detoxification in MOH: results of a 12-month detoxification study. Cephalalgia.

[53] Diener HC, Antonaci F, Braschinsky M, Evers S, Jensen R, Lainez M, Kristoffersen ES, Tassorelli C, Ryliskiene K, Petersen JA (2020). European academy of neurology guideline on the management of medication-overuse headache. Eur J Neurol.

[54] Katsarava Z, Fritsche G, Muessig M, Diener HC, Limmroth V (2001). Clinical features of withdrawal headache following overuse of triptans and other headache drugs. Neurology.

[55] Kristoffersen ES, Straand J, Vetvik KG, Benth JŠ, Russell MB, Lundqvist C (2015). Brief intervention for medication-overuse headache in primary care. The BIMOH study: a double-blind pragmatic cluster randomised parallel controlled trial. J Neurol Neurosurg Psychiatry.

[56] Katsarava Z, Muessig M, Dzagnidze A, Fritsche G, Diener HC, Limmroth V (2005). Medication overuse headache: rates and predictors for relapse in a 4-year prospective study. Cephalalgia.

[57] Carlsen LN, Munksgaard SB, Nielsen M, Engelstoft IMS, Westergaard ML, Bendtsen L, Jensen RH (2020). Comparison of 3 treatment strategies for medication overuse headache: a randomized clinical trial. JAMA Neurol.

[58] Young NP, Elrashidi MY, McKie PM, Ebbert JO (2018). Neuroimaging utilization and findings in headache outpatients: significance of red and yellow flags. Cephalalgia.

[59] Do TP, Remmers A, Schytz HW, Schankin C, Nelson SE, Obermann M, Hansen JM, Sinclair AJ, Gantenbein AR, Schoonman GG (2019). Red and orange flags for secondary headaches in clinical practice: SNNOOP10 list. Neurology.

[60] Kamins J, Charles A (2018). Posttraumatic headache: basic mechanisms and therapeutic targets. Headache.

[61] Mollan SP, Aguiar M, Evison F, Frew E, Sinclair AJ (2019). The expanding burden of idiopathic intracranial hypertension. Eye (Lond).

[62] Amrhein TJ, Kranz PG (2019). Spontaneous intracranial hypotension: imaging in diagnosis and treatment. Radiol Clin N Am.

[63] Sharma A, Mohammad AJ, Turesson C (2020). Incidence and prevalence of giant cell arteritis and polymyalgia rheumatica: a systematic literature review. Semin Arthritis Rheum.

[64] Kristoffersen ES, Harper CE, Vetvik KG, Zarnovicky S, Hansen JM, Faiz KW (2020). Incidence and mortality of cerebral venous thrombosis in a Norwegian population. Stroke.

[65] McKinney PA (2004). Brain tumours: incidence, survival, and aetiology. J Neurol Neurosurg Psychiatry.

[66] Sharp HJ, Denman D, Puumala S, Leopold DA (2007). Treatment of acute and chronic rhinosinusitis in the United States, 1999-2002. Arch Otolaryngol Head Neck Surg.

[67] Di SG, Maarbjerg S, Nurmikko T (2018). Triggering trigeminal neuralgia. Cephalalgia.

[68] Maarbjerg S, Wolfram F, Heinskou TB, Rochat P, Gozalov A, Brennum J, Olesen J, Bendtsen L (2017). Persistent idiopathic facial pain - a prospective systematic study of clinical characteristics and neuroanatomical findings at 3.0 tesla MRI. Cephalalgia.

[69] Peker S, Dincer A, Necmettin PM (2009). Vascular compression of the trigeminal nerve is a frequent finding in asymptomatic individuals: 3-T MR imaging of 200 trigeminal nerves using 3D CISS sequences. Acta Neurochir.

[70] Devor M, Amir R, Rappaport ZH (2002). Pathophysiology of trigeminal neuralgia: the ignition hypothesis. Clin J Pain.

[71] Bendtsen L, Zakrzewska JM, Abbott J, Braschinsky M, di Stefano G, Donnet A, Eide PK, Leal PRL, Maarbjerg S, May A, Nurmikko T, Obermann M, Jensen TS, Cruccu G (2019). European academy of neurology guideline on trigeminal neuralgia. Eur J Neurol.

[72] Jensen TS (2002). Anticonvulsants in neuropathic pain: rationale and clinical evidence. Eur J Pain.

[73] Bendtsen L, Zakrzewska JM, Heinskou TB, Hodaie M, Leal PRL, Nurmikko T, Obermann M, Cruccu G, Maarbjerg S (2020). Advances in diagnosis, classification, pathophysiology, and management of trigeminal neuralgia. Lancet Neurol.

[74] Heinskou TB, Rochat P, Maarbjerg S, Wolfram F, Brennum J, Olesen J, Bendtsen L (2019). Prognostic factors for outcome of microvascular decompression in trigeminal neuralgia: a prospective systematic study using independent assessors. Cephalalgia.

[75] Maasumi K, Tepper SJ, Kriegler JS (2017). Menstrual migraine and treatment options: review. Headache.

[76] Mahmoud AN, Mentias A, Elgendy AY, Qazi A, Barakat AF, Saad M, Mohsen A, Abuzaid A, Mansoor H, Mojadidi MK, Elgendy IY (2018). Migraine and the risk of cardiovascular and cerebrovascular events: a meta-analysis of 16 cohort studies including 1 152 407 subjects. BMJ Open.

[77] Adelborg K, Szepligeti SK, Holland-Bill L (2018). Migraine and risk of cardiovascular diseases: Danish population based matched cohort study. BMJ.

[78] Sacco S, Merki-Feld GS, AEgidius KL (2017). Hormonal contraceptives and risk of ischemic stroke in women with migraine: a consensus statement from the European headache federation (EHF) and the European Society of Contraception and Reproductive Health (ESC). J Headache Pain.

[79] Monteith TS, Gardener H, Rundek T, Elkind MSV, Sacco RL (2015). Migraine and risk of stroke in older adults: northern Manhattan study. Neurology.

[80] Sacco S, Merki-Feld GS, AEgidius KL (2018). Effect of exogenous estrogens and progestogens on the course of migraine during reproductive age: a consensus statement by the European headache federation (EHF) and the European Society of Contraception and Reproductive Health (ESCRH). J Headache Pain.

[81] Nierenburg HC, Ailani J, Malloy M (2015). Systematic review of preventive and acute treatment of menstrual migraine. Headache.

[82] Calhoun AH (2017). Migraine treatment in pregnancy and lactation. Curr Pain Headache Rep.

[83] Maunuksela EL, Olkkola KT, Korpela R (1987). Measurement of pain in children with self-reporting and behavioral assessment. Clin Pharmacol Ther.

[84] Hamalainen ML, Hoppu K, Valkeila E, Santavuori P (1997). Ibuprofen or acetaminophen for the acute treatment of migraine in children: a double-blind, randomized, placebo-controlled, crossover study. Neurology.

[85] Lewis DW, Kellstein D, Dahl G, Burke B, Frank LM, Toor S, Northam RS, White LW, Lawson L (2002). Children's ibuprofen suspension for the acute treatment of pediatric migraine. Headache.

[86] Barbanti P, Grazzi L, Egeo G (2019). Pharmacotherapy for acute migraines in children and adolescents. Expert Opin Pharmacother.

[87] Bakhshandeh BM, Rahbarimanesh AA, Sadeghi M (2015). Comparison of propranolol and pregabalin for prophylaxis of childhood migraine: a randomised controlled trial. Acta Med Iran.

[88] Stubberud A, Flaaen NM, McCrory DC, Pedersen SA, Linde M (2019). Flunarizine as prophylaxis for episodic migraine: a systematic review with meta-analysis. Pain.

[89] Hershey AD, Powers SW, Coffey CS, Eklund DD, Chamberlin LA, Korbee LL, CHAMP Study Group (2013). Childhood and adolescent migraine prevention (CHAMP) study: a double-blinded, placebo-controlled, comparative effectiveness study of amitriptyline, topiramate, and placebo in the prevention of childhood and adolescent migraine. Headache.

